# Bidirectional associations between mental health conditions and cognitive impairment in patients with pain conditions of the back, neck, and spine: A population-based study

**DOI:** 10.1371/journal.pone.0352339

**Published:** 2026-06-23

**Authors:** Mohammad Alipour-Vaezi, Jyoti Savla, Margaret R. Rukstalis, Daniel B. Rukstalis, Kwok-Leung Tsui, Donald B. Penzien, Robert S. McNamara, Huaiyang Zhong

**Affiliations:** 1 Grado Department of Industrial & Systems Engineering, Virginia Tech, Blacksburg, Virginia, United States of America; 2 Department of Human Development and Family Science, Virginia Tech, Blacksburg, Virginia, United States of America; 3 Department of Psychiatry and Behavioral Medicine, Virginia Tech Carilion School of Medicine, Roanoke, Virginia, United States of America; 4 Department of Surgery, Virginia Tech Carilion School of Medicine, Roanoke, Virginia, United States of America; 5 Department of Industrial, Manufacturing, and Systems Engineering, University of Texas at Arlington, Arlington, Texas, United States of America; 6 Departments of Psychiatry and Behavioral Medicine, Neurology, & Epidemiology and Prevention, Wake Forest University School of Medicine, Winston Salem, North Carolina, United States of America; Mayo Clinic Florida, UNITED STATES OF AMERICA

## Abstract

**Background:**

Pain conditions (PCs) of the back, neck, and spine are frequently accompanied by psychiatric and cognitive comorbidities in older adults. However, the directionality and magnitude of the associations between psychiatric disorders and cognitive impairment in this population remain insufficiently characterized.

**Objective:**

This population-based study examines the bidirectional relationship between Mental Health Conditions (MHCs) and Cognitive Impairment (CI) among patients with back, neck, and spine pain. It assesses whether MHCs—including depression, Bipolar Disorder (BD), Generalized Anxiety Disorder (GAD), Post-Traumatic Stress Disorder (PTSD), Panic Disorder (PaD), Persistent Mood Disorder (PMD), Suicidal Behavior (SB), Schizophrenia (SCZ), and Substance Use Disorder (SUD)—are associated with subsequent incident CI, and conversely, whether prior CI is associated with subsequent incident MHCs.

**Methods:**

Data were drawn from the TriNetX US Collaborative Network (2016/01/01–2021/12/31), comprising over 119 million patients. Cohorts of patients with PCs were defined using ICD-10 codes. Propensity score matching was applied to balance demographics and comorbidities. Kaplan-Meier survival analysis assessed risks over a three-year follow-up.

**Results:**

A bidirectional association was observed between MHCs and CI. PC Patients with MHCs had a higher three-year risk for CI, with the largest risk ratios (RR) observed for SCZ (RR: 4.594; 95% Confidence Interval [3.974, 5.312]) and BD (RR: 3.761[3.247, 4.356]). Other MHCs, including depression, PMD, GAD, PTSD, PaD, and SUD, were also associated with higher CI risk. Conversely, patients with pre-existing CI exhibited a higher three-year risk for subsequent MHCs, particularly BD (RR: 4.818 [3.045, 7.624]), SCZ (RR: 3.398 [2.874, 4.017]) and SB (RR: 1.913 [1.340, 2.732]).

**Conclusion:**

Our findings indicate a bidirectional relationship between MHCs and CI among older adults with documented back, neck, and spine pain. Integrated screening and coordinated multidisciplinary care may help identify psychiatric and cognitive comorbidities earlier and support more comprehensive management of this medically complex population.

## 1. Introduction

Pain conditions (PCs) are highly prevalent among adults and are associated with substantial physical and psychological health, and quality of life burden [1,2]. Chronic pain, characterized by pain persisting for over 12 weeks [3], represents an especially burdensome subset of PCs. A 2021 study indicated that approximately 21% of adults in the U.S. experienced chronic pain [4]. National Center for Health Statistics data revealed that 60% of adults reported pain in the last three months, with back pain being the most prevalent, affecting 39% of respondents [5]. Given the high prevalence and clinical burden of back, neck, and spine pain, understanding cognitive and psychiatric comorbidities among older adults with documented PCs in these anatomical regions is particularly important.

Mounting evidence links chronic pain and pain-related conditions to Cognitive Impairment (CI) in the form of cognitive deficits and increased risk for neurocognitive disorders and dementia [6] (the operational definition of CI for this study is delineated in section 2.2). Adults with high-impact chronic pain demonstrate an 18% prevalence of CI versus 3% in those who are pain-free [7], and 65% of individuals with CI report pain issues, including 46% of individuals with Alzheimer’s disease [8]. Specific pain syndromes, such as chronic lower-back pain, are often associated with poor memory and attention [9], and pre-operative CI in one-fifth of spine-surgery candidates predicts worse postoperative outcomes [10,11].

Mental Health Conditions (MHCs) are also commonly associated with chronic pain and pain-related conditions [12,13]. Approximately 35–45% of individuals with chronic pain also experienced depression [14,15], and 25% of individuals with chronic pain report symptoms of anxiety and/or depression, fivefold higher than the population without chronic pain [16]. Co-occurrence also extends to Post-Traumatic Stress Disorder (PTSD) and Bipolar Disorder (BD) [17,18]. Mood and anxiety disorders, in turn, are associated with greater pain intensity, disability, and health-care utilization [19]. Furthermore, a meta-analysis identified a high prevalence of Suicidal Behavior (SB), including suicidal ideation and suicide attempts, among people with chronic pain [20].

Several overlapping pathophysiological and clinical pathways may help explain why chronic pain, MHCs, and CI frequently co-occur. Persistent or recurrent pain has been associated with alterations in brain regions involved in attention, memory, emotional regulation, and pain modulation, including prefrontal-limbic and thalamocortical networks [21–24]. Pain-related symptoms may also contribute to neuroinflammation, hypothalamic-pituitary-adrenal axis dysregulation, autonomic arousal, sleep disturbance, and reduced physical activity, all of which have been linked to both psychiatric symptoms and cognitive decline [25–28]. Conversely, MHCs may be associated with altered pain perception through altered affective processing, attentional bias toward pain, catastrophizing, reduced coping capacity, and impaired treatment adherence [29–31]. CI may be associated with additional pain-management challenges, including limitations in medication adherence, self-monitoring, communication of symptoms, and engagement with behavioral or rehabilitative interventions [32–34]. These shared pathways support the clinical rationale for examining MHC-CI associations specifically among older adults with documented PCs of the back, neck, and spine.

Despite these epidemiological and pathophysiological connections, less is known about how MHCs and CI are associated with each other among patients with documented back, neck, and spine PCs. Cross-sectional studies document cognitive deficit across a range of mental illnesses [35–37], and evidence suggests that MHCs have a greater impact on cognitive decline than many somatic diseases or demographic factors [38]. Yet prior work has typically examined only one directional pathway- either MHC to CI or CI to MHC- and often relies on small single-center samples, limiting generalizability. Even fewer investigations focus on patients already burdened by back, neck, and spine PCs, despite emerging evidence that convergent biological and psychosocial mechanisms may be associated with greater neuropsychiatric vulnerability [39–41]. As a result, clinicians still lack reliable, population-level estimates of how mental-health diagnoses and cognitive deficits co-occur or appear sequentially within this large and clinically fragile group. Because back, neck, and spine pain-related conditions are associated with both psychiatric morbidity and cognitive vulnerability, we examined MHC–CI bidirectional associations specifically within this PC population rather than in a general adult cohort [42,43].

To close this knowledge gap, we leveraged the TriNetX U.S. Collaborative Network, comprising 119 million de-identified electronic health records from 68 healthcare organizations, to assemble a retrospective cohort of adults aged 65 years or older with newly documented PCs of the back, neck, and spine between 2016 and 2021. After propensity-score matching for demographics and comorbidities, we pursued two complementary objectives: (i) to quantify the association between pre-existing MHCs and the three-year incidence estimates for CI, and (ii) to quantify the association between pre-existing CI and the three-year incidence estimates for MHCs in the same population. Characterizing these reciprocal associations may inform integrated screening, referral, and multidisciplinary treatment strategies for older adults living with documented back, neck, and spine PCs.

## 2. Methods

### 2.1. Data source and study population

In this research, a retrospective cohort study was conducted with data from the TriNetX US Collaborative Network [44,45]. TriNetX is a federated real-world data and analytics platform that provides access to de-identified EHR-derived data contributed by participating healthcare organizations. The platform contains structured clinical information, including patient demographics, encounters, diagnoses, procedures, medications, laboratory results, and other routinely documented clinical variables [46]. The dataset used in this study represents approximately 119 million patients affiliated with 68 healthcare organizations in the United States and includes records from both inpatient and outpatient settings, such as hospitals, primary care clinics, and specialist clinics. TriNetX Analytics allows investigators to define cohorts using standardized clinical codes, apply inclusion and exclusion criteria, conduct cohort comparisons, perform propensity-score matching, and estimate risk-based and time-to-event outcomes [47]. TriNetX deidentification complies with the standards in Section 164.514(a) of the HIPAA Privacy Rule, verified by a qualified expert’s formal determination per Section 164.512(b)(1) [48]. This manuscript reports a retrospective study of medical records that were fully anonymized before being accessed by the research team (TriNetX database). IRB designation of this study as non-human subjects research was obtained by the research team (IRB-24–1741). The Carilion Clinic IRB reviewed this study and determined it does not meet the definition of human subjects research (45 CFR 46.102(d)); therefore, the IRB did not require approval or oversight of this study.

TriNetX demographics and diagnoses reflect structured EHR documentation. We did not perform imputation. For categorical demographics (e.g., race/ethnicity/sex) that may be missing in EHR data, TriNetX can display these values as “Unknown”; patients with unknown categories were retained, and such categories were treated as valid levels when included in baseline covariates. Comorbidities were defined based on the presence of diagnosis codes; absence of a coded diagnosis was treated as absence of the corresponding recorded comorbidity, recognizing that under-coding or care received outside participating systems may result in misclassification.

To maintain patient confidentiality, the TriNetX system automatically adjusted the number of patients to 10 where outcomes involved fewer than 10 patients in either cohort. Statistical comparisons of continuous and categorical data were conducted using t-tests and chi-square tests, respectively, with significance set at the level of 0.05. All statistical analyses were performed using the TriNetX Analytics tool, integrated within the TriNetX platform.

### 2.2. Study cohorts

**Fig 1:**
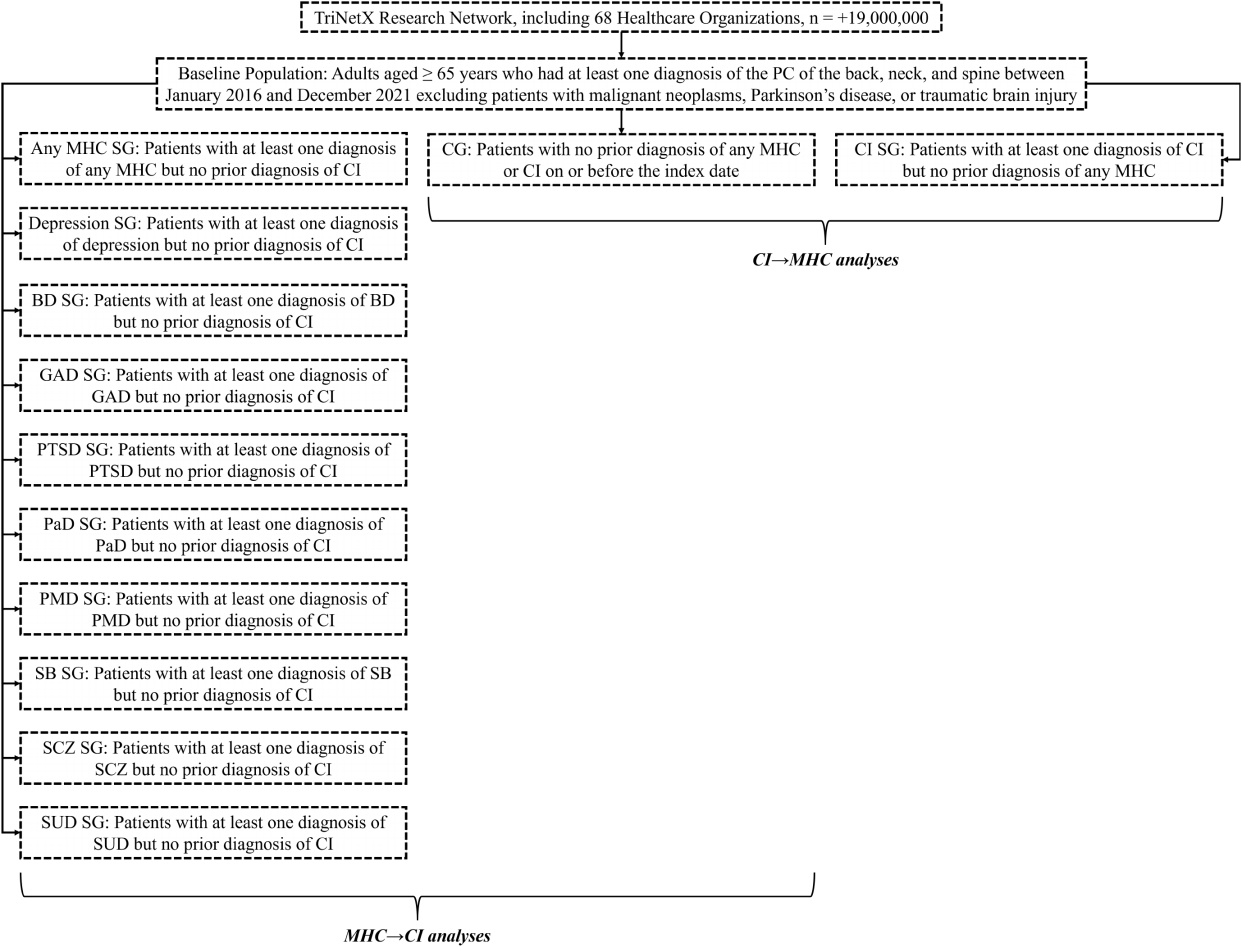
Flowchart of cohort construction. SG: Study Group; CG: Control Group; MHC: Mental Health Condition; BD: Bipolar Disorder; PTSD: Post-traumatic Stress Disorder; GAD: Generalized Anxiety Disorder; PaD: Panic Disorder; PMD: Persistent Mood Disorder; SB: Suicidal Behavior; SCZ: Schizophrenia; SUD: Substance Use; PC: Pain Conditions; CI: Cognitive Impairment.

To construct study cohorts, the TriNetX database was accessed using the International Classification of Diseases, Tenth Revision (ICD-10) codes to meet the criteria for diagnoses. Table 1 represents the ICD-10 code(s) used as the baseline for each diagnosis. Data was collected for visits to the network hospitals from January 1, 2016, to December 31, 2021. Inclusion and exclusion criteria that were applied across each cohort are depicted in Fig 1.

**Table 1:** Baseline Diagnosis and Related ICD-10 Codes.

Diagnosis		ICD-10/RxNorm
Pain Conditions of the Back, Neck, and Spine	Cervical spine pain	M50
	Neck and back pain	M54.2-M54.6; M54.89; M54.9
	Radiculopathy	M54.1
	Spinal Stenosis	M48; M99.23; M99.33; M99.43; M99.53; M99.63 M99.73
	Thoracic, Thoracolumbar, and Lumbosacral Intervertebral Disc Disorders	M51
	Fibromyalgia	M79.7
	Spondylosis	M47
Metabolic and vascular Conditions	Type 1 Diabetes Mellitus	E10
	Type 2 Diabetes Mellitus	E11
	Overweight and obesity	E66
	Hyperlipidemia	E78
	Essential hypertension	I10
Cardiovascular Diseases	Coronary artery/ischemic heart disease	I25; Z95.1
	Acute myocardial infarction	I21
	Heart failure	I50
	Atrial fibrillation/flutter	I48
	Peripheral arterial disease	I70; Z95.820
Cerebrovascular Diseases	Ischaemic stroke	I63
	Haemorrhagic stroke	I60; I61
	Transient ischaemic attack	G45
	Other cerebrovascular disease	I67
Chronic Lower Respiratory Disease	Bronchitis	J40-J42
	Emphysema	J43
	Chronic Obstructive Pulmonary Disease	J44
	Asthma	J45-J46
	Bronchiectasis	J47
Chronic Kidney Disease		N18
Sepsis		A40; A41
Mental Health Condition	Depression	F32-F33
	Bipolar Disorder	F31
	Generalized Anxiety Disorder	F41.1
	Post-Traumatic Stress Disorder	F43.1
	Panic disorder	F41.0
	Persistent mood disorder	F34
	Suicidal Behavior	T14.91; R45.851
	Schizophrenia	F20-F29
	Substance Use Disorder	F10-F19
Cognitive Impairment		F01-F03; F06.7; G30.0; G30.1; G30.8; G30.9; G31.0; G31.1
Exclusion Conditions	Malignant neoplasms (cancer)	C00–C97
	Parkinson’s disease	G20
	Traumatic Brain Injury	S06

This timeframe allows for a 3-year follow-up period to assess the incidence of CI and MHCs, considering that CI typically develops over time. Only patients who were ≥ 65 years old at the index PC diagnosis of the back, neck, and spine were included in this study. This age restriction was used to focus on an older population in whom CI is more commonly diagnosed and clinically consequential, thereby improving interpretability for geriatric care and reducing heterogeneity associated with substantially different baseline CI risk profiles in younger adults.

Baseline (pre-existing) CI and MHC status were defined using the presence of the corresponding ICD-10 diagnosis codes recorded on or before the index date (Table 1). Diagnosis codes were used for phenotype definition; medications were not used as proxies for CI or MHC status. Because TriNetX is a multi-site EHR network rather than a claims database with continuous enrollment records, available pre-index lookback may vary across patients, and diagnoses received outside participating healthcare organizations may not be captured. No minimum prior observation period was required in the primary analysis. Therefore, incident outcomes in this study should be interpreted as newly documented EHR diagnoses after the index date rather than definitive biological onset of disease.

Clinical Concepts for Orthopedics was used as the basis for selecting codes representing PCs of the back, neck, and spine [49]. We limited PCs to back, neck, and spine diagnoses to define a clinically coherent and highly prevalent musculoskeletal pain phenotype in older adults and to reduce heterogeneity that would arise from pooling fundamentally different PC etiologies with distinct clinical courses, coding practices, and risk profiles for MHCs and CI. Code selection was accomplished with input from medical specialists familiar with these diagnoses. MHCs commonly co-occurring with PCs of the back, neck, and spine, per previous literature, were included in the study, and representative ICD-10 codes were selected with clinical expert input.

CI was operationalized using ICD-10 codes F01–F03, F06.7, G30.0, G30.1, G30.8, G30.9, G31.0, and G31.1 (Table 1). This definition was selected to capture persistent neurocognitive disorders documented in structured EHR diagnosis fields, including dementia diagnoses (F01–F03; G30.*; G31.0–G31.1) and mild neurocognitive disorder (F06.7). During code selection, we excluded diagnoses representing transient/acute cognitive states (e.g., delirium) and cognitive symptoms explicitly attributable to acute intoxication/withdrawal or other clearly delineated secondary causes, to focus on persistent CI diagnoses rather than short-lived syndromes. In addition, patients with malignant neoplasms, Parkinson’s disease, and Traumatic Brain Injury were excluded to reduce potential confounding and ensure cohort specificity.

MHCs were operationalized using ICD-10 codes for depression (F32–F33), BD (F31), GAD (F41.1), PTSD (F43.1), PaD (F41.0), PMD (F34), SCZ and related psychotic disorders (F20–F29), and SUD (F10–F19) (Table 1). These code sets were selected to capture clinician-diagnosed psychiatric conditions documented in structured EHR diagnosis fields rather than symptom-only presentations. To reduce heterogeneity and misclassification, we did not use nonspecific psychiatric symptom codes or broad “unspecified” categories as substitutes for disorder-level diagnoses. SB was identified using T14.91 and R45.851 (Table 1) and was analyzed as a clinically important construct; although SB is not itself an MHC diagnosis, it is strongly associated with underlying psychiatric illness and was therefore included as a separate study group and within the “Any MHC” composite definition.

The Control Group (CG) cohort was defined once and used as a shared reference group in both directions of analysis. It comprised all patients with documented PCs of the back, neck, and spine who had no recorded MHC and no recorded CI on or before the index date. All Study Groups (SGs) were propensity-score matched to this same CG to estimate incident outcomes during follow-up.

For the MHC → CI analyses, nine SGs were constructed. Each included patients with documented PCs of the back, neck, and spine who had a prior diagnosis of one specific MHC but no prior CI. These disorders were: depression, BD, Generalized Anxiety Disorder (GAD), PTSD, Panic Disorder (PaD), Persistent Mood Disorder (PMD) (includes cyclothymia, dysthymia, unspecified mood disorders), SB (while not a MHC, the large majority of people attempting suicide have a MHC [50]), Schizophrenia (SCZ), and Substance Use Disorder (SUD). In addition, an “Any mental health condition (Any MHC)” cohort pooled patients with documented PCs of the back, neck, and spine with any of the psychiatric disorders evaluated in this study or SB, again excluding those with prior CI. This terminology was used because SB is clinically relevant to mental health but is not itself a psychiatric diagnosis. These groups enabled us to examine the associations between individual and aggregate MHCs and subsequent CI risk.

For the CI → MHC analyses, we built an additional SG consisting of patients with documented PCs of the back, neck, and spine who had a documented CI diagnosis but no MHC diagnosis on or before the index date. We compared this SG with the same CG described above to quantify the association between pre-existing CI and the incidence of MHCs.

Outcomes were defined as incident diagnoses occurring after the index date and within a standardized 3-year follow-up period. CI was defined as the first occurrence after the index date of any CI in the TriNetX database during follow-up among patients without those CI codes on or before the index date. MHCs were defined analogously by the first documented diagnosis of MHC codes after the index PC diagnosis among patients without those MHC codes on or before the index date.

Because ICD-10 codes reflect clinical documentation rather than standardized research assessments, outcome definitions represent coded clinical diagnoses and may be subject to under-documentation or misclassification inherent to EHR data. Therefore, this study captured recognized and documented diagnoses of PCs of the back, neck, and spine, MHCs, and CI. Unrecognized, undiagnosed, or undocumented conditions could not be identified if patients were not evaluated for these conditions or if the relevant ICD-10 codes were not recorded in the EHR.

We used a 3-year follow-up window to ensure a consistent and comparable observation period across all cohorts and both directions of the bidirectional analysis, while balancing outcome ascertainment against loss to follow-up and heterogeneity in longitudinal capture inherent to multi-site EHR-derived data. Accordingly, time-to-event analyses were censored at 3 years after the index date.

To address the possibility that a single qualifying ICD-10 diagnosis could capture acute or subacute pain, we conducted two sensitivity analyses using stricter chronicity-oriented definitions of the PC phenotype. In the first sensitivity analysis, PC was defined as at least two diagnoses within the same qualifying PC diagnosis category from the prespecified back, neck, and spine ICD-10 code listed in Table 1, with the two diagnoses recorded 3–6 months apart. For example, a patient qualified if they had two qualifying diagnoses for the same PC category, such as two cervical spine pain diagnoses, two neck/back pain diagnoses, two radiculopathy diagnoses, or two spinal stenosis-related diagnoses within the specified time window. This approach was intended to strengthen the operational definition of chronicity while preserving the clinically curated PC categories used in the primary analysis. In the second sensitivity analysis, PC was defined using a chronicity-enriched ICD-10 subset including M50, M48, M99.23, M99.33, M99.43, M99.53, M99.63, M99.73, M51, M79.7, and M47. For both sensitivity analyses, the primary composite comparisons were repeated: prior Any MHC→incident CI and prior CI→incident Any MHC.

### 2.3. Statistical analysis

Propensity score matching [51,52] was employed to match patients in each of the two CGs to those in the related SGs in a 1:1 ratio. Propensity scores, ranging from 0 to 1, were calculated using logistic regression to predict the membership in the baseline cohorts based on patient covariates, including demographics—age, gender, and race—and medical conditions such as cardiovascular diseases, cerebrovascular diseases, metabolic and vascular conditions, chronic lower respiratory disease, chronic kidney disease, and sepsis. The greedy nearest neighbor algorithm was used for matching [53].

After propensity score matching, relative risks and Kaplan–Meier survival curves [54] were estimated using the TriNetX analytics platform after propensity score matching on selected baseline covariates. The primary analyses were defined as the two main bidirectional composite comparisons: (i) the association between prior Any MHC and subsequent incident CI, and (ii) the association between prior CI and subsequent incident Any MHC. The two primary composite comparisons were repeated under both stricter chronicity-oriented PC sensitivity definitions described above.

To assess the potential influence of delayed documentation of pre-existing disease, we performed a simple back-of-the-envelope robustness assessment using the matched cohort counts reported in Tables 2 and 3. For each primary comparison and selected secondary comparisons, we estimated the number and proportion of post-index outcome diagnoses in the SG that would need to represent delayed documentation rather than newly documented outcomes for the observed excess risk to disappear. This was calculated as the difference between the observed number of outcome cases in the SG and the observed number of outcome cases in the matched CG, divided by the observed number of outcome cases in the SG.

**Table 2:** Cognitive Impairment differences between Patients with PCs with different Mental Health Conditions after 3-year follow-up.

MHC	Study Group		Control Group		Risk Ratio	95% Confidence Interval	ARD (%)
**Incident Cases (N)**	**Risk (%)**	**Incident Cases (N)**	**Risk (%)**
Any MHC	12,912	2.8	7,266	1.6	1.777	(1.727, 1.828)	+1.2
Depression	8,345	3.3	4,457	1.7	1.872	(1.806, 1.941)	+1.6
BD	835	3.5	222	0.9	3.761	(3.247, 4.356)	+2.6
GAD	1,727	2.9	958	1.6	1.803	(1.667, 1.949)	+1.3
PTSD	239	1.8	101	0.7	2.366	(1.878, 2.982)	+1.1
PaD	247	2.2	153	1.4	1.614	(1.322, 1.972)	+0.8
PMD	444	3.0	210	1.4	2.114	(1.797, 2.487)	+1.6
SB	281	3.8	88	1.2	3.193	(2.519, 4.048)	+2.6
SCZ	997	6.4	217	1.4	4.594	(3.974, 5.312)	+5.0
SUD	4,343	2.0	2,543	1.2	1.708	(1.627, 1.793)	+0.8

MHC: Mental Health Condition; BD: Bipolar Disorder; PTSD: Post-traumatic Stress Disorder; GAD: Generalized Anxiety Disorder; PaD: Panic Disorder; PMD: Persistent Mood disorder; SB: Suicidal Behavior; SCZ: Schizophrenia; SUD: Substance Use; ARD: Absolute Risk Difference.

**Table 3:** The risk ratio of developing different Mental Health Conditions among Patients with PCs with a prior cognitive impairment diagnosis within a 3-year follow-up period (After matching).

MHC	Study Group		Control Group		Risk Ratio	95% Confidence Interval	ARD (%)
**Incident Cases (N)**	**Risk (%)**	**Incident Cases (N)**	**Risk (%)**
Any MHC	4,426	12.1	2,946	8.1	1.502	(1.437, 1.570)	+4.0
Depression	3,195	8.7	1,963	5.4	1.628	(1.542, 1.718)	+3.3
BD	106	0.3	22	0.1	4.818	(3.045, 7.624)	+0.2
GAD	319	0.9	308	0.8	1.036	(0.886, 1.210)	+0.1
PTSD	35	0.1	27	0.1	1.296	(0.785, 2.141)	+0.0
PaD	56	0.2	67	0.2	0.836	(0.586, 1.192)	+0.0
PMD	102	0.3	78	0.2	1.308	(0.974, 1.755)	+0.1
SB	88	0.2	46	0.1	1.913	(1.340, 2.732)	+0.1
SCZ	598	1.6	176	0.5	3.398	(2.874, 4.017)	+1.1
SUD	595	1.6	683	1.9	0.871	(0.781, 0.971)	−0.3

MHC: Mental Health Condition; BD: Bipolar Disorder; PTSD: Post-traumatic Stress Disorder; GAD: Generalized Anxiety Disorder; PaD: Panic Disorder; PMD: Persistent Mood disorder; SB: Suicidal Behavior; SCZ: Schizophrenia; SUD: Substance Use; ARD: Absolute Risk Difference.

Disorder-specific analyses were treated as secondary/exploratory analyses to characterize which psychiatric diagnostic categories contributed most to the overall bidirectional pattern. Accordingly, disorder-specific findings, particularly those with small absolute risk differences or confidence intervals close to the null, were interpreted cautiously.

These analyses assume that the matched cohorts are comparable with respect to observed characteristics (exchangeability), that exposures and outcomes are accurately captured by diagnosis codes, and that follow-up censoring is independent of the event process. Kaplan–Meier estimation further assumes correct event timing and non-informative censoring. While propensity score matching reduces confounding by observed covariates, residual confounding due to unmeasured factors may remain.

## 3. Results

### 3.1. Demographic and population characteristics

A total of 1,843,483 individuals were initially identified as the Control Group (CG). Baseline demographics for both the CG and each SG cohort for analysis of the associations between PC, MHCs, and CI are detailed in S1 Table. Propensity score matching was conducted across various cohorts, including 492,103 patients identified with any MHC, 260,272 with depression, 23,897 with BD, 60,219 with GAD, 13,551 with PTSD, 11,160 with PaD, 14,755 with PMD, 7,335 with SB, 15,544 with SCZ, and 222,567 with SUD. Furthermore, an additional cohort was created to investigate the association between prior CI and the incidence of MHCs among patients with documented PCs of the back, neck, and spine. This cohort includes 36,560 patients with documented PCs of the back, neck, and spine with prior CI diagnosis. S2 Table lists the baseline demographics for both the CG and each SG cohort for analysis of the association between CI and MHC.

Before matching, several demographic and clinical differences were observed between the CG and SGs. For example, compared with the CG, patients with any prior MHC had higher proportions of type 2 diabetes mellitus, hyperlipidemia, essential hypertension, chronic lower respiratory disease, chronic kidney disease, and cardiovascular/cerebrovascular comorbidities. Similar baseline differences were observed in several disorder-specific cohorts; for instance, patients with prior depression, BD, SCZ, and SUD generally showed higher comorbidity burdens than the CG. In the CI → MHC analysis, patients with prior CI were substantially older than the CG and had higher frequencies of vascular and cardiometabolic comorbidities, including essential hypertension, atrial fibrillation/flutter, ischemic stroke, chronic kidney disease, and sepsis. To address these baseline differences and improve comparability between cohorts, we applied 1:1 propensity-score matching on age, sex, race, and major comorbidity groups before estimating outcome risks. Detailed pre- and post-matching cohort characteristics are presented in S1–S13 Tables.

### 3.2. Association of prior mental health conditions with cognitive impairment incidence

For the primary MHC → CI analysis, prior Any MHC was associated with a higher three-year incidence estimate for subsequent CI compared with the matched control group without prior MHC or CI (RR = 1.777; 95% confidence interval: 1.727, 1.828; Table 2). In secondary disorder-specific analyses, the largest relative risk estimates were observed for SCZ (RR = 4.594; 95% confidence interval: 3.974, 5.312), BD (RR = 3.761; 95% confidence interval: 3.247, 4.356), and SB (RR = 3.193; 95% confidence interval: 2.519, 4.048). Other MHCs, including depression, GAD, PTSD, PaD, PMD, and SUD, were also statistically associated with higher incident CI risk, with complete disorder-specific estimates presented in Table 2.

To aid the interpretation of the effect size magnitude, Table 2 reports both relative risks (risk ratios) and the corresponding absolute 3-year risks (%). Across matched comparisons, the control-group 3-year CI risk ranged from 0.7% to 1.7%. For example, the SCZ cohort had a 6.4% 3-year CI risk versus 1.4% in its matched control group (absolute risk difference +5.0 percentage points (pp); RR 4.594). The BD and SB cohorts showed 3.5% vs 0.9% (+2.6 pp; RR 3.761) and 3.8% vs 1.2% (+2.6 pp; RR 3.193), respectively. In contrast, more modest RRs corresponded to smaller absolute differences (e.g., PaD 2.2% vs 1.4%, + 0.8 pp; SUD 2.0% vs 1.2%, + 0.8 pp), which may have more limited standalone clinical implications.

**Fig 2:**
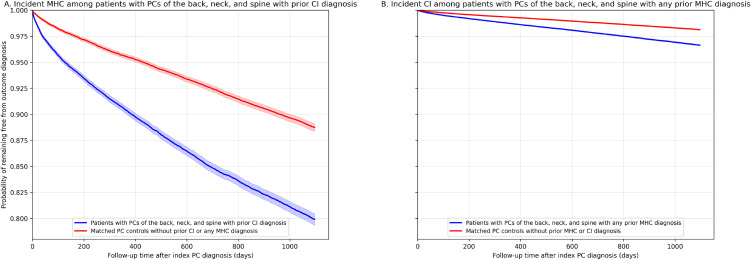
Kaplan–Meier event-free survival curves for incident mental health conditions and incident cognitive impairment during the 3-year follow-up period among patients with pain conditions of the back, neck, and spine. Panel A shows incident MHC among Patients with PCs with prior CI compared with matched PC controls without prior CI or MHC. Panel B shows incident CI among Patients with PCs with prior Any MHC compared with matched PC controls without prior MHC or CI. The x-axis represents follow-up time after the index PC diagnosis in days, and the y-axis represents the estimated probability of remaining free from the outcome diagnosis. MHC: Mental Health Condition; PC: Pain Condition; CI: Cognitive Impairment.

Kaplan–Meier analysis for the MHC → CI comparison (Fig 2B) showed that Patients with PCs with prior Any MHC had a lower probability of remaining free from incident CI during follow-up than matched PC controls without prior MHC or CI. The three-year event-free survival probability was 96.6% in the prior Any MHC cohort compared with 98.1% in the matched CG. Cox proportional hazards analysis showed a similar pattern (hazard ratio: 1.811; 95% confidence interval: 1.760, 1.864).

### 3.3. Association of prior cognitive impairment with mental health condition incidence

For the primary CI → MHC analysis, prior CI was associated with a higher three-year incidence estimate for subsequent Any MHC compared with the matched control group without prior CI or MHC (RR = 1.502; 95% confidence interval: 1.437, 1.570; Table 3). In secondary disorder-specific analyses, the largest relative risk estimates were observed for BD (RR = 4.818; 95% confidence interval: 3.045, 7.624), SCZ (RR = 3.398; 95% confidence interval: 2.874, 4.017), SB (RR = 1.913; 95% confidence interval: 1.340, 2.732), and depression (RR = 1.628; 95% confidence interval: 1.542, 1.718). Other disorder-specific estimates were smaller or not statistically significant and are presented in Table 3. These secondary findings should be interpreted as exploratory, particularly where absolute risk differences were small.

Table 3 similarly presents absolute risks and risk ratios for incident MHCs following baseline CI. Several estimates close to the null corresponded to very small absolute differences, despite statistical testing in a large cohort (e.g., GAD 0.9% vs 0.8%, + 0.1 pp, RR 1.036; PMD 0.3% vs 0.2%, + 0.1 pp, RR 1.308; PaD 0.2% vs 0.2%, approximately 0.0 pp, RR 0.836). By contrast, larger absolute differences were observed for any MHC (12.1% vs 8.1%, + 4.0 pp, RR 1.502) and depression (8.7% vs 5.4%, + 3.3 pp, RR 1.628). We also note that large relative risks for rare outcomes can translate into small absolute differences (e.g., BD 0.3% vs 0.1%, + 0.2 pp, RR 4.818), underscoring the importance of reporting absolute measures alongside relative measures.

The outcomes presented above were limited to individual MHCs and any MHC. We did not include combinations such as depression and GAD or depression and PTSD due to the limitations of the TriNetX database in tracking the incidence of multiple MHCs simultaneously following a diagnosis of CI.

Kaplan–Meier analysis for the CI → MHC comparison (Fig 2A) showed that Patients with PCs with prior CI had a lower probability of remaining free from incident MHC during follow-up than matched PC controls without prior CI or MHC. The three-year event-free survival probability was 80.1% in the prior-CI cohort compared with 89.1% in the matched CG. Cox proportional hazards analysis showed a similar pattern (hazard ratio: 2.007; 95% confidence interval: 1.916, 2.103).

### 3.4. Sensitivity and robustness analyses

First, sensitivity analyses using stricter PC definitions showed results consistent with the primary analysis. When PC was defined as at least two diagnoses within the same qualifying PC category recorded 3–6 months apart, the MHC → CI analysis showed a risk ratio of 1.944 (95% CI: 1.897, 1.992), and the CI → MHC analysis showed a risk ratio of 1.487 (95% CI: 1.381, 1.601). When PC was defined using only chronicity-enriched ICD-10 codes, the MHC → CI analysis showed a risk ratio of 1.877 (95% CI: 1.821, 1.934), and the CI → MHC analysis showed a risk ratio of 1.251 (95% CI: 1.191, 1.314). These findings suggest that the main bidirectional pattern was not driven solely by patients with a single potentially acute or subacute back, neck, or spine pain diagnosis.

Second, to assess the potential influence of incomplete prior-history capture, we performed a back-of-the-envelope robustness assessment using the matched cohort counts in Tables 2 and 3. In the overall MHC → CI analysis, 12,912 CI cases were observed in the Any MHC cohort compared with 7,266 in the matched CG; therefore, approximately 5,646 cases, or 43.7% of observed CI cases in the Any MHC cohort, would need to reflect delayed documentation to reduce the association to the null. For the strongest MHC → CI findings, the corresponding proportions were approximately 78.2% for SCZ, 73.4% for BD, and 68.7% for SB. In the CI → MHC analysis, 4,426 MHC cases were observed in the prior-CI cohort compared with 2,946 in the matched CG; therefore, approximately 1,480 cases, or 33.4% of observed MHC cases in the prior-CI cohort, would need to reflect delayed documentation to fully explain the overall CI → MHC association. These calculations do not eliminate the possibility of incomplete prior-history capture, but they suggest that substantial differential delayed documentation would be required to fully account for the main observed associations.

## 4. Discussion

This study examined the bidirectional associations between MHCs and CI among older adults with documented PCs of the back, neck, and spine using a large retrospective EHR-based cohort. Building on prior evidence linking pain-related conditions, MHCs, and CI, we aimed to advance the literature by examining the bidirectional associations between MHCs and CI among older adults with PC of the back, neck, and spine. Overall, our results indicate that the presence of several MHCs—including depression, BD, GAD, PTSD, PaD, PMD, SB, SCZ, and SUD—was significantly associated with elevated three-year risk estimates for incident CI in patients with PC. Importantly, documented PCs of the back, neck, and spine should be understood as the clinical context in which these bidirectional associations were examined. Older adults with back, neck, and spine PCs may already experience pain-related functional limitation, sleep disturbance, reduced physical activity, and increased healthcare contact, all of which may intersect with psychiatric and cognitive vulnerability [55–58]. Therefore, the observed MHC–CI associations should be interpreted within the broader clinical complexity of chronic back, neck, and spine pain. The sensitivity analyses using repeated PC-category diagnoses and a chronicity-enriched ICD-10 subset further supported the overall bidirectional pattern under stricter chronicity-oriented definitions. Additionally, these patients with documented PCs of the back, neck, and spine and pre-existing CI had higher three-year risk estimates for several subsequent MHCs compared with matched patients without prior CI or MHCs. The importance of recognizing these comorbidities is underscored by existing literature showing that psychiatric comorbidities are associated with more complex pain experience, functional outcomes, and clinical management in patients with PC [59–62]. In the present study, this concern is further reinforced by the observed bidirectional associations between prior MHCs and subsequent CI, and between prior CI and subsequent MHCs, among older adults with PC of the back, neck, and spine.

In the MHC → CI analysis, the presence of SCZ, BD, SB, PTSD, and PMD was associated with the highest CI risk ratios among patients with documented PCs of the back, neck, and spine. These results are consistent with previous research outlining associations between PC and MHCs with regard to impairments in various cognitive domains, including memory and attention [63–65]. Similarly, the established presence and importance of cognitive dysfunction in patients with MHCs is also reinforced in this research [66]. Notably, patients with documented PCs of the back, neck, and spine and pre-existing SCZ demonstrated the highest three-year risk estimate for incident CI. Indeed, the association between SCZ and CI is well documented, including evidence of greater cognitive decline and neurovascular burden among patients with SCZ [65]. Additionally, our findings align with recent literature demonstrating strong evidence for a connection between serious mental illness and premature aging, with possible relevance to susceptibility to neurocognitive disorders [67]. Evidence also exists for multifactorial connections between BD and CI [68]. The association observed for SB should be interpreted cautiously, because SB is not itself a psychiatric diagnosis but is closely linked to underlying psychiatric illness, mood and anxiety disorders, and dementia-related vulnerability [69–71].

In the CI → MHC analysis, the risk ratios for subsequent SCZ and BD were notably high, with BD showing the highest relative risk among the psychiatric outcomes evaluated among patients with documented PCs of the back, neck, and spine and pre-existing CI. One potential explanation for this finding is some similarity in symptom presentation; for example, a portion of patients with frontotemporal dementia are misdiagnosed or concurrently diagnosed with BD or SCZ [72–74]. The risk for developing SB was also significantly elevated, and depression remained substantially increased among patients with PCs and prior CI. The observed CI → MHC associations appeared strongest for psychiatric outcomes with recognized cognitive or neuropsychiatric features. One possible explanation is that prodromal neurocognitive symptoms may later be documented as part of a late-onset MHC. Conversely, SUD showed lower risk ratios in our research, a finding counter to a strong body of evidence supporting cognitive deficits as a risk factor for SUD [75]. The age of our study population (65 years or older) may account for this difference, as psychosocial variables for older adults with PC and CI, such as the need for caregiver involvement, may be reflected in lower observed diagnosis rates of substance use concerns.

Kaplan-Meier analysis indicated that patients with documented PCs of the back, neck, and spine and pre-existing CI were more likely to receive a subsequent MHC diagnosis during follow-up than the matched CG (80.1% vs. 89.0%). We also found a 1.5% difference in the three-year incidence estimates for subsequent CI over three years for patients with PC and MHCs compared to CG. These findings should be interpreted by considering both relative and absolute measures of effect. In several disorder-specific analyses, relatively high risk ratios occurred in the context of low baseline event rates; therefore, large relative risks may correspond to modest absolute risk differences. Clinically, these findings remain meaningful because they help identify subgroups of older adults with documented back, neck, and spine PCs who may warrant closer psychiatric or cognitive attention during follow-up. However, small absolute risk differences should be interpreted cautiously and should not be overstated as large individual-level risk increases. Thus, the results support integrated screening and coordinated care while also emphasizing the importance of interpreting statistical significance alongside absolute clinical magnitude.

These findings have important clinical and public health implications for the management of older adults with PC of the back, neck, and spine. Because prior MHCs were associated with subsequent CI and prior CI was associated with subsequent MHCs, psychiatric and cognitive conditions should be considered as interrelated comorbidities in this population. In practice, this supports multimorbidity-aware care models in which pain management, psychiatric evaluation, and cognitive screening are coordinated rather than addressed separately. For patients with PC and documented MHCs, clinicians may consider closer attention to cognitive symptoms during follow-up. Similarly, for patients with PC and documented CI, psychiatric symptoms may warrant earlier recognition and referral. These implications do not establish causal effects but highlight the value of integrated screening and coordinated care pathways for a clinically complex group of older adults.

### 4.1. Strengths and limitations

The primary strength of this study is the large, multisite cohort, which includes patients from diverse clinical settings and enhances the generalizability of the findings. Second, the use of 1:1 propensity score matching is a methodological strength that improves covariate balance and cohort comparability, thereby reducing confounding from unmeasured variables and strengthening the interpretability of the observed associations. In addition, the use of real-world data from existing medical records enhances external validity. However, reliance on clinician diagnosis and ICD-10 coding is also a limitation, as diagnostic rigor and coding practices cannot be directly controlled [76]. When defining diagnostic condition categories using ICD-10 codes, excluding broad or nonspecific codes may reduce confounding but may also exclude patients with clinical relevant histories. For example, excluding anxiety disorder, unspecified, from this study likely removes patients with anxiety resulting from PC of the back, neck, and spine, but also patients who may have GAD. Although sensitivity analyses used repeated PC-category diagnoses and a chronicity-enriched ICD-10 subset, EHR-based ICD-10 definitions cannot fully capture pain duration, intensity, anatomical severity, or functional impact. Thus, findings should be interpreted as associations among patients with documented back, neck, and spine PCs diagnoses rather than directly measured pain chronicity or severity.

Incomplete capture of prior history is another limitation of multi-site EHR data. Patients may have received CI or MHC diagnoses outside participating healthcare organizations, and the absence of a pre-index ICD-10 code in TriNetX does not necessarily confirm the absence of disease. Although our back-of-the-envelope robustness assessment suggested that a substantial proportion of post-index diagnoses would need to represent delayed documentation to fully explain the main associations, this calculation does not replace a formal washout analysis and cannot fully eliminate the possibility of incomplete prior-history capture. We also could not distinguish whether each qualifying diagnosis was recorded in an outpatient or inpatient setting. Because encounter setting may affect diagnostic certainty, disease severity, and surveillance intensity, this limitation should be considered when interpreting findings.

Detection or surveillance bias is also an important limitation. Patients with MHCs, CI, or greater medical complexity may have more frequent encounters and therefore more opportunities for additional diagnoses to be detected and recorded. The current TriNetX interface available to us did not provide a direct and reliable encounter-count variable that could be incorporated as a healthcare-utilization proxy in the present analysis. Adjustment for encounter frequency would require exported patient-level data and additional data-governance approval, which was not feasible within the current revision timeline. Therefore, differences in diagnostic intensity may have contributed to the observed associations and should be considered when interpreting the results.

Although our study was carefully designed to include patients with documented PCs of the back, neck, and spine without prior diagnoses to assess incidence over the subsequent three years, the findings remain correlational and should not be interpreted causally. Given the large sample size, even small differences can reach statistical significance; therefore, the clinical relevance of modest associations, partically RRs with confidence interval near 1, should be interpreted with caution. Follow-up duration is an additional limitation. CI and dementia-related diagnoses may develop over longer intervals than 3 years, particularly in a cohort restricted to patients aged ≥65 years. Therefore, restricting follow-up to 3 years may underestimate longer-term cumulative incidence and may not fully capture slower trajectories of cognitive decline. Our findings should be interpreted as associations within a 3-year post-index window, and future studies should evaluate these associations over longer horizons (e.g., 5–10 years) and/or in prospective cohorts with repeated, standardized cognitive assessments.

Competing risks, particularly death, are also important in this older cohort. Standard Kaplan–Meier and Cox approaches assume non-informative censoring; however, death may preclude subsequent documentation of CI or MHC and may therefore act as a competing event. Without a formal competing-risk analysis, the direction of potential bias cannot be determined with certainty. If patients with prior MHCs or CI have higher mortality than matched controls, treating death as non-informative censoring may overestimate cumulative incidence in the exposed cohorts and potentially exaggerate relative associations. Conversely, if death occurs before CI or MHC can be documented, observed diagnosis rates may be underestimated, potentially attenuating the associations. Accordingly, our time-to-event estimates should be interpreted as associations with documented diagnoses during follow-up rather than competing-risk-adjusted cumulative incidence estimates.

Another limitation of this study is the lack of a specified time frame for assessing prior MHCs in patients with PC. We chose not to impose such a restriction to capture a wider range of psychiatric conditions, including those that may have developed episodically or over a longer period, which could still be related to cognitive function and pain perception. Furthermore, residual and unmeasured confounding is likely. Several important determinants of both MHCs and CI—particularly education level (cognitive reserve), socioeconomic status, mobility/functional status, and physical activity—are not consistently available as structured variables in TriNetX and therefore could not be comprehensively adjusted for. Also, TriNetX diagnosis codes do not encode domain-specific neurocognitive profiles (e.g., memory, executive function, or language), and structured neuropsychological testing data are not consistently captured in EHR-derived networks; consequently, we could not determine which cognitive domain(s) were impaired, which limits the granularity of interpretation of CI-related findings. In addition, psychiatric and pain-related medications (e.g., antidepressants, antipsychotics, mood stabilizers, benzodiazepines, opioids, and adjuvant analgesics) may serve as proxies for underlying disease severity or treatment indication; however, medication exposure is not captured uniformly or reliably across participating health systems in a way that permits comprehensive confounding adjustment. Accordingly, our estimates should be interpreted as associations that may be influenced by residual/unmeasured confounding despite matching and adjustment for measured covariates.

Future studies could address these limitations through prospective designs incorporating standardized measures of cognition (including domain-specific testing), pain severity and interference, educational attainment, and mobility/physical activity assessments. EHR-based analyses could further evaluate robustness through (i) adjustment for available proxy indicators of functional impairment/frailty (e.g., fall-related diagnoses, use of assistive devices, frailty-related codes) and (ii) baseline adjustment/stratification by medication exposure classes when medication data are sufficiently complete. These approaches would help clarify the extent to which the observed associations are explained by cognitive reserve, functional limitation, socioeconomic status, or medication-related confounding. Complementary designs, such as negative control outcomes or exposures, alternative cohort definitions requiring repeated pain encounters, and replication across independent networks, could further strengthen interpretability and generalizability. Future work should also examine whether directly measured pain duration, intensity, anatomical severity, functional impact, encounter settings, and years lived with PC, MHCs, or CI before the index date modify the observed MHC-CI associations. Finally, detailed cross-tabulation and subgroup analyses by age, sex, and other clinically relevant characteristics to determine whether the observed associations differ across patient subgroups.

### 4.2. Conclusions

In this study, we observed a clear, bidirectional relationship between MHCs and CI among older adults with documented PCs of the back, neck, and spine: pre-existing MHCs—particularly SCZ, BD, and SB—were associated with higher subsequent CI risk, while prior CI was associated with subsequent MHC risk. By quantifying both directions in a single, real-world dataset, our study fills a critical evidence gap and underscores the need for integrated care pathways that span primary care, pain management, psychiatry, and geriatrics. For example, integrated pain management care teams, while pooling expertise to mitigate patients’ experience of pain, could benefit from early targeted screening of psychiatric and cognitive concerns, thus capturing potential undiagnosed comorbidities and informing treatment. In clinics without integrated models, these results suggest the value of brief, cross-specialty screening and facilitating referrals to specialized care. Considering that assessment and effective multidisciplinary treatment approaches can be cumbersome and time-consuming processes for all involved, future research may focus on broadening resources (1) to streamline screening to expedite appropriate referrals and treatment, and (2) to improve collaboration and communication between members of treatment teams for these medically complex patients.

## Supporting information

S1 Table Baseline Demographic Characteristics for Patients with PCs with or without prior diagnosis of different Mental Health Conditions. BD: Bipolar Disorder; PTSD: Post-traumatic Stress Disorder; GAD: Generalized Anxiety Disorder; PaD: Panic Disorder; PMD: Persistent Mood disorder; SB: Suicidal Behavior; SCZ: Schizophrenia; SUD: Substance Use Disorder; CKD: Chronic Kidney Disease; CLRD: Chronic Lower Respiratory Disease; CVD: Cardiovascular Diseases; CBVC: Cerebrovascular Diseases; MVC: Metabolic and vascular Conditions; *: Presented in Number (Percentage of Cohort) format; **: Presented in Mean (Standard Deviation) format.
(PDF)

S2 Table Baseline Demographic Characteristics for Patients with PCs with or without prior diagnosis of cognitive impairment. BD: Bipolar Disorder; PTSD: Post-traumatic Stress Disorder; GAD: Generalized Anxiety Disorder; PaD: Panic Disorder; PMD: Persistent Mood disorder; SB: Suicidal Behavior; SCZ: Schizophrenia; SUD: Substance Use Disorder; CKD: Chronic Kidney Disease; CLRD: Chronic Lower Respiratory Disease; CVD: Cardiovascular Diseases; CBVC: Cerebrovascular Diseases; MVC: Metabolic and vascular Conditions; *: Presented in Number (Percentage of Cohort) format; **: Presented in Mean (Standard Deviation) format.
(PDF)

S3 Table Baseline Demographic Characteristics for Patients with Pain Conditions with any Mental Health Condition after Propensity Score Matching. BD: Bipolar Disorder; PTSD: Post-traumatic Stress Disorder; GAD: Generalized Anxiety Disorder; PaD: Panic Disorder; PMD: Persistent Mood disorder; SB: Suicidal Behavior; SCZ: Schizophrenia; SUD: Substance Use Disorder; CKD: Chronic Kidney Disease; CLRD: Chronic Lower Respiratory Disease; CVD: Cardiovascular Diseases; CBVD: Cerebrovascular Diseases; MVC: Metabolic and Vascular Conditions; *: Presented in Number (Percentage of Cohort) format; **: Presented in Mean (Standard Deviation) format.
(PDF)

S4 Table Baseline Demographic Characteristics for Patients with Pain Conditions with Depression after Propensity Score Matching. BD: Bipolar Disorder; PTSD: Post-traumatic Stress Disorder; GAD: Generalized Anxiety Disorder; PaD: Panic Disorder; PMD: Persistent Mood disorder; SB: Suicidal Behavior; SCZ: Schizophrenia; SUD: Substance Use Disorder; CKD: Chronic Kidney Disease; CLRD: Chronic Lower Respiratory Disease; CVD: Cardiovascular Diseases; CBVD: Cerebrovascular Diseases; MVC: Metabolic and vascular Conditions; *: Presented in Number (Percentage of Cohort) format; **: Presented in Mean (Standard Deviation) format.
(PDF)

S5 Table Baseline Demographic Characteristics for Patients with Pain Conditions with Bipolar Disorder after Propensity Score Matching. BD: Bipolar Disorder; PTSD: Post-traumatic Stress Disorder; GAD: Generalized Anxiety Disorder; PaD: Panic Disorder; PMD: Persistent Mood disorder; SB: Suicidal Behavior; SCZ: Schizophrenia; SUD: Substance Use Disorder; CKD: Chronic Kidney Disease; CLRD: Chronic Lower Respiratory Disease; CVD: Cardiovascular Diseases; CBVD: Cerebrovascular Diseases; MVC: Metabolic and vascular Conditions; *: Presented in Number (Percentage of Cohort) format; **: Presented in Mean (Standard Deviation) format.
(PDF)

S6 Table Baseline Demographic Characteristics for Patients with Pain Conditions with Generalized Anxiety Disorder after Propensity Score Matching. BD: Bipolar Disorder; PTSD: Post-traumatic Stress Disorder; GAD: Generalized Anxiety Disorder; PaD: Panic Disorder; PMD: Persistent Mood disorder; SB: Suicidal Behavior; SCZ: Schizophrenia; SUD: Substance Use Disorder; CKD: Chronic Kidney Disease; CLRD: Chronic Lower Respiratory Disease; CVD: Cardiovascular Diseases; CBVD: Cerebrovascular Diseases; MVC: Metabolic and vascular Conditions; *: Presented in Number (Percentage of Cohort) format; **: Presented in Mean (Standard Deviation) format.
(PDF)

S7 Table Baseline Demographic Characteristics for Patients with Pain Conditions with Post-Traumatic Stress Disorder after Propensity Score Matching. BD: Bipolar Disorder; PTSD: Post-traumatic Stress Disorder; GAD: Generalized Anxiety Disorder; PaD: Panic Disorder; PMD: Persistent Mood disorder; SB: Suicidal Behavior; SCZ: Schizophrenia; SUD: Substance Use Disorder; CKD: Chronic Kidney Disease; CLRD: Chronic Lower Respiratory Disease; CVD: Cardiovascular Diseases; CBVD: Cerebrovascular Diseases; MVC: Metabolic and vascular Conditions; *: Presented in Number (Percentage of Cohort) format; **: Presented in Mean (Standard Deviation) format.
(PDF)

S8 Table Baseline Demographic Characteristics for Patients with Pain Conditions with Panic Disorder after Propensity Score Matching. BD: Bipolar Disorder; PTSD: Post-traumatic Stress Disorder; GAD: Generalized Anxiety Disorder; PaD: Panic Disorder; PMD: Persistent Mood disorder; SB: Suicidal Behavior; SCZ: Schizophrenia; SUD: Substance Use Disorder; CKD: Chronic Kidney Disease; CLRD: Chronic Lower Respiratory Disease; CVD: Cardiovascular Diseases; CBVD: Cerebrovascular Diseases; MVC: Metabolic and vascular Conditions; *: Presented in Number (Percentage of Cohort) format; **: Presented in Mean (Standard Deviation) format.
(PDF)

S9 Table Baseline Demographic Characteristics for Patients with Pain Conditions with Persistent Mood Disorder after Propensity Score Matching. BD: Bipolar Disorder; PTSD: Post-traumatic Stress Disorder; GAD: Generalized Anxiety Disorder; PaD: Panic Disorder; PMD: Persistent Mood disorder; SB: Suicidal Behavior; SCZ: Schizophrenia; SUD: Substance Use Disorder; CKD: Chronic Kidney Disease; CLRD: Chronic Lower Respiratory Disease; CVD: Cardiovascular Diseases; CBVD: Cerebrovascular Diseases; MVC: Metabolic and vascular Conditions; *: Presented in Number (Percentage of Cohort) format; **: Presented in Mean (Standard Deviation) format.
(PDF)

S10 Table Baseline Demographic Characteristics for Patients with Pain Conditions with Suicidal Behavior after Propensity Score Matching. BD: Bipolar Disorder; PTSD: Post-traumatic Stress Disorder; GAD: Generalized Anxiety Disorder; PaD: Panic Disorder; PMD: Persistent Mood disorder; SB: Suicidal Behavior; SCZ: Schizophrenia; SUD: Substance Use Disorder; CKD: Chronic Kidney Disease; CLRD: Chronic Lower Respiratory Disease; CVD: Cardiovascular Diseases; CBVD: Cerebrovascular Diseases; MVC: Metabolic and vascular Conditions; *: Presented in Number (Percentage of Cohort) format; **: Presented in Mean (Standard Deviation) format.
(PDF)

S11 Table Baseline Demographic Characteristics for Patients with Pain Conditions with Schizophrenia after Propensity Score Matching. BD: Bipolar Disorder; PTSD: Post-traumatic Stress Disorder; GAD: Generalized Anxiety Disorder; PaD: Panic Disorder; PMD: Persistent Mood disorder; SB: Suicidal Behavior; SCZ: Schizophrenia; SUD: Substance Use Disorder; CKD: Chronic Kidney Disease; CLRD: Chronic Lower Respiratory Disease; CVD: Cardiovascular Diseases; CBVD: Cerebrovascular Diseases; MVC: Metabolic and vascular Conditions; *: Presented in Number (Percentage of Cohort) format; **: Presented in Mean (Standard Deviation) format.
(PDF)

S12 Table Baseline Demographic Characteristics for Patients with Pain Conditions with Substance Use Disorder after Propensity Score Matching. BD: Bipolar Disorder; PTSD: Post-traumatic Stress Disorder; GAD: Generalized Anxiety Disorder; PaD: Panic Disorder; PMD: Persistent Mood disorder; SB: Suicidal Behavior; SCZ: Schizophrenia; SUD: Substance Use Disorder; CKD: Chronic Kidney Disease; CLRD: Chronic Lower Respiratory Disease; CVD: Cardiovascular Diseases; CBVD: Cerebrovascular Diseases; MVC: Metabolic and vascular Conditions; *: Presented in Number (Percentage of Cohort) format; **: Presented in Mean (Standard Deviation) format.
(PDF)

S13 Table Baseline Demographic Characteristics for Patients with Pain Conditions with Cognitive Impairment after Propensity Score Matching. BD: Bipolar Disorder; PTSD: Post-traumatic Stress Disorder; GAD: Generalized Anxiety Disorder; PaD: Panic Disorder; PMD: Persistent Mood disorder; SB: Suicidal Behavior; SCZ: Schizophrenia; SUD: Substance Use Disorder; CKD: Chronic Kidney Disease; CLRD: Chronic Lower Respiratory Disease; CVD: Cardiovascular Diseases; CBVD: Cerebrovascular Diseases; MVC: Metabolic and vascular Conditions; *: Presented in Number (Percentage of Cohort) format; **: Presented in Mean (Standard Deviation) format.
(PDF)
