# Supplementary material for: Bidirectional associations between mental health conditions and cognitive impairment in patients with pain conditions of the back, neck, and spine: A population-based study
Source: PLoS One. 2026 Jun 23;21(6):e0352339. doi: 10.1371/journal.pone.0352339 (PMC13289910; doi:10.1371/journal.pone.0352339)
Supplement: S1 Table — BD: Bipolar Disorder; PTSD: Post-traumatic Stress Disorder; GAD: Generalized Anxiety Disorder; PaD: Panic Disorder; PMD: Persistent Mood disorder; SB: Suicidal Behavior; SCZ: Schizophrenia; SUD: Substance Use Disorder; CKD: Chronic Kidney Disease; CLRD: Chronic Lower Respiratory Disease; CVD: Cardiovascular Diseases; CBVC: Cerebrovascular Diseases; MVC: Metabolic and vascular Conditions; *: Presented in Number (Percentage of Cohort) format; **: Presented in Mean (Standard Deviation) format. (PDF) [file pone.0352339.s001.pdf]

Table S1. Baseline Demographic Characteristics for Patients with pain conditions with or without prior diagnosis of different Mental helth conditions. BD: Bipolar Disorder; PTSD: Post-traumatic Stress Disorder; GAD: Generalized Anxiety Disorder; PaD: Panic Disorder; PMD: Persistent Mood disorder; SB: Suicidal Behavior; SCZ: Schizophrenia; SUD: Substance Use Disorder; CKD: Chronic Kidney Disease; CLRD: Chronic Lower Respiratory Disease; CVD: Cardiovascular Diseases; CBVC: Cerebrovascular Diseases; MVC: Metabolic and vascular Conditions; *: Presented in Number (Percentage of Cohort) format; **: Presented in Mean (Standard Deviation) format.

Characteristic		Control Group	Study Group									
			Any Mental helth condition	Depression	BD	GAD	PTSD	PaD	PMD	SB	SCZ	SUD
Total Population*		1,843,483 (100)	492,103 (100)	260,272 (100)	23,897 (100)	60,219 (100)	13,551 (100)	11,160 (100)	14,755 (100)	7,335 (100)	15,544 (100)	222,567 (100)
Age**		69.8 (8.5)	67.1 (7.7)	67.9 (8.1)	64.6 (6.2)	67.8 (8.2)	64.8 (6.1)	66.3 (7.5)	67.2 (8.0)	64.8 (7.0)	66.0 (7.5)	65.5 (6.7)
Sex*	Male	761,358 (41.3)	183,062 (37.2)	43,878 (72.8)	7,767 (32.5)	15,235 (25.3)	5,881 (43.4)	2,957 (26.5)	3,660 (49.9)	3,660 (49.9)	6,777 (43.6)	113,954 (51.2)
	Female	1,080,281 (58.6)	308,548 (62.7)	16,334 (27.1)	16,106 (67.4)	44,983 (74.7)	7,656 (56.5)	8,203 (73.5)	3,675 (50.1)	3,675 (50.1)	8,751 (56.3)	108,613 (48.8)
	Unknown	1,840 (0.1)	492 (0.1)	59 (0.1)	24 (0.1)	10 (0.0)	11 (0.1)	11 (0.1)	10 (0.1)	10 (0.1)	16 (0.1)	222 (0.1)
Race*	White	1,342,055 (72.8)	355,790 (72.3)	48,880 (81.1)	18,567 (77.7)	50,704 (84.2)	10.448 (77.1)	9,174 (82.2)	5,575 (76.0)	5,575 (76.0)	9,466 (60.9)	159,803 (71.8)
	Black	223,061 (12.1)	68,402 (13.9)	6,329 (10.5)	3,441 (14.4)	4,757 (7.9)	1,707 (12.6)	982 (8.8)	1,093 (14.9)	1,093 (14.9)	4,352 (28.0)	42,510 (19.1)
	Asian	86,643 (4.7)	8,366 (1.7)	843 (1.4)	215 (0.9)	1,024 (1.7)	203 (1.5)	223 (2.0)	125 (1.7)	125 (1.7)	358 (2.3)	4,006 (1.8)
	Other	60,834 (3.3)	36,907 (7.5)	1,386 (2.3)	478 (2.0)	1,204 (2.0)	407 (3.0)	257 (2.3)	176 (2.4)	176 (2.4)	373 (2.4)	5,564 (2.5)
	Unknown	130,887 (7.1)	22,636 (4.6)	2,832 (4.7)	1194 (5.0)	2,529 (4.2)	786 (5.8)	525 (4.7)	367 (5.0)	367 (5.0)	995 (6.4)	10,683 (4.8)
MVC*	Type 1 Diabetes Mellitus	28,219 (1.5)	18,738 (3.8)	12,114 (4.7)	1,015 (4.2)	2,553 (4.2)	510 (3.8)	395 (3.5)	286 (3.9)	286 (3.9)	844 (5.4)	7,080 (3.2)
	Type 2 Diabetes Mellitus	339,437 (18.4)	144,914 (29.4)	83,854 (32.2)	7,751 (32.4)	15,788 (26.2)	4,252 (31.4)	2,997 (26.9)	2,233 (30.4)	2,233 (30.4)	5,740 (36.9)	61,059 (27.4)
	Overweight and obesity	226,812 (12.3)	122,226 (24.8)	77,016 (29.6)	6,815 (28.5)	15,290 (25.4)	3,878 (28.6)	3,221 (28.9)	1,547 (21.1)	1,547 (21.1)	4,111 (26.4)	44,873 (20.2)
	Hyperlipidemia	730,961 (39.7)	298,203 (60.6)	172,037 (66.1)	13,657 (57.1)	41,753 (69.3)	7,872 (58.1)	7,387 (66.2)	3,503 (47.8)	3,503 (47.8)	8,542 (55.0)	119,410 (53.7)
	Essential hypertension	824,595 (44.7)	333,758 (67.8)	183,268 (70.4)	15,521 (64.9)	42,334 (70.3)	8,789 (64.9)	7,518 (67.4)	4,839 (66.0)	4,839 (66.0)	10,847 (69.8)	147,745 (66.4)
CVD*	Coronary artery/ischemic heart disease	269,531 (14.6)	129,805 (26.4)	69,200 (26.6)	5,429 (22.8)	14,088 (23.4)	3,593 (26.5)	2,805 (25.1)	3,360 (22.7)	1,995 (27.2)	3,789 (24.4)	65,345 (29.3)
	Acute myocardial infarction	42,123 (2.3)	26,937 (5.5)	13,733 (5.3)	1,084 (4.5)	2,562 (4.3)	765 (5.6)	660 (5.9)	465 (6.3)	465 (6.3)	912 (5.9)	15,164 (6.8)
	Heart failure	99,339 (5.4)	61,388 (12.5)	35,336 (13.6)	2,920 (12.2)	6,455 (10.7)	1,616 (11.9)	1,396 (12.5)	1,021 (13.9)	1,021 (13.9)	2,509 (16.1)	29,485 (13.2)
	Atrial fibrillation/flutter	136,705 (7.4)	52,374 (10.6)	30,083 (11.6)	1,946 (8.1)	6,048 (10.0)	1,322 (9.8)	1,183 (10.6)	752 (10.3)	752 (10.3)	1,630 (10.5)	22,616 (10.2)
	Peripheral arterial disease	69,183 (3.7)	38,944 (7.9)	19,124 (7.4)	1,368 (5.7)	4,322 (7.2)	731 (5.4)	788 (7.1)	970 (6.6)	447 (6.1)	1,090 (7.1)	22,181 (10.0)
CBVC*	Ischaemic stroke	46,553 (2.5)	28,103 (5.7)	15,511 (6.0)	1,277 (5.3)	3,098 (5.1)	750 (5.5)	566 (5.1)	440 (6.0)	440 (6.0)	1,198 (7.7)	13,662 (6.1)
	Haemorrhagic stroke	6,988 (0.3)	4,313 (0.9)	2,444 (1.0)	176 (0.7)	372 (0.7)	111 (0.8)	80 (0.7)	113 (0.8)	66 (0.9)	137 (0.9)	2,185 (1.0)
	Transient ischaemic attack	35,044 (1.9)	17,832 (3.6)	10,433 (4.0)	874 (3.7)	2,672 (4.4)	486 (3.6)	527 (4.7)	207 (2.8)	207 (2.8)	665 (4.3)	7,294 (3.3)
	Other cerebrovascular disease	43,996 (2.4)	25,014 (5.1)	14,322 (5.5)	1,257 (5.3)	3,307 (5.5)	621 (4.6)	618 (5.5)	418 (5.7)	418 (5.7)	1,258 (8.1)	10,995 (4.9)
CLRD*		241,911 (13.1)	176,833 (35.9)	92,537 (35.6)	9,652 (40.4)	21,475 (35.7)	5,347 (39.5)	4,255 (38.1)	5,199 (35.2)	2,639 (36.0)	6,178 (39.7)	92,869 (41.7)
CKD*		133,272 (7.2)	64,301 (13.1)	38,236 (14.7)	3,563 (14.9)	7,401 (12.3)	1,622 (12.0)	1,323 (11.9)	1,800 (12.2)	959 (13.1)	2,621 (16.9)	26,120 (11.7)
Sepsis*		162,173 (1.7)	26,555 (5.4)	14,731 (5.7)	1,562 (6.6)	2,580 (4.3)	721 (5.3)	563 (5.1)	607 (4.1)	579 (7.8)	1,571 (10.1)	14,199 (6.0)
