# Supplementary material for: Bidirectional associations between mental health conditions and cognitive impairment in patients with pain conditions of the back, neck, and spine: A population-based study
Source: PLoS One. 2026 Jun 23;21(6):e0352339. doi: 10.1371/journal.pone.0352339 (PMC13289910; doi:10.1371/journal.pone.0352339)
Supplement: S2 Table — BD: Bipolar Disorder; PTSD: Post-traumatic Stress Disorder; GAD: Generalized Anxiety Disorder; PaD: Panic Disorder; PMD: Persistent Mood disorder; SB: Suicidal Behavior; SCZ: Schizophrenia; SUD: Substance Use Disorder; CKD: Chronic Kidney Disease; CLRD: Chronic Lower Respiratory Disease; CVD: Cardiovascular Diseases; CBVC: Cerebrovascular Diseases; MVC: Metabolic and vascular Conditions; *: Presented in Number (Percentage of Cohort) format; **: Presented in Mean (Standard Deviation) format. (PDF) [file pone.0352339.s002.pdf]

Table S2. Baseline Demographic Characteristics for Patients with pain conditions with or without prior diagnosis of cognitive impairment. BD: Bipolar Disorder; PTSD: Post-traumatic Stress Disorder; GAD: Generalized Anxiety Disorder; PaD: Panic Disorder; PMD: Persistent Mood disorder; SB: Suicidal Behavior; SCZ: Schizophrenia; SUD: Substance Use Disorder; CKD: Chronic Kidney Disease; CLRD: Chronic Lower Respiratory Disease; CVD: Cardiovascular Diseases; CBVC: Cerebrovascular Diseases; MVC: Metabolic and vascular Conditions; *: Presented in Number (Percentage of Cohort) format; **: Presented in Mean (Standard Deviation) format.

Characteristic		Control Group	Study Group
Total Population*		1,843,483 (100)	36,560 (100)
Age**		69.8 (8.5)	82.6 (7.1)
Sex*	Male	761,358 (41.3)	12,211 (33.4)
	Female	1,080,281 (58.6)	813,255 (66.6)
	Unknown	1,840 (0.1)	10 (0.1)
Race*	White	1,342,055 (72.8)	26,469 (72.4)
	Black	223,061 (12.1)	4,241 (11.6)
	Asian	86,643 (4.7)	2,523 (6.9)
	Other	60,834 (3.3)	951 (2.6)
	Unknown	130,887 (7.1)	2,376 (6.5)
MVC*	Type 1 Diabetes Mellitus	28,219 (1.5)	832 (2.3)
	Type 2 Diabetes Mellitus	339,437 (18.4)	10,722 (29.3)
	Overweight and obesity	226,812 (12.3)	3,136 (8.6)
	Hyperlipidemia	730,961 (39.7)	19,950 (54.6)
	Essential hypertension	824,595 (44.7)	25,554 (69.9)
CVD*	Coronary artery/ischemic heart disease	269,531 (14.6)	12,126 (33.2)
	Acute myocardial infarction	42,123 (2.3)	2,484 (6.8)
	Heart failure	99,339 (5.4)	7,345 (20.1)
	Atrial fibrillation/flutter	136,705 (7.4)	8,776 (24.0)
	Peripheral arterial disease	69,183 (3.7)	3,519 (9.6)
CBVC*	Ischaemic stroke	46,553 (2.5)	4,066 (11.1)
	Haemorrhagic stroke	6,988 (0.3)	625 (1.7)
	Transient ischaemic attack	35,044 (1.9)	2,026 (5.5)
	Other cerebrovascular disease	43,996 (2.4)	4,588 (12.5)
CLRD*		241,911 (13.1)	7,029 (19.2)
CKD*		133,272 (7.2)	8,431 (23.1)
Sepsis*		30,305 (1.7)	3,565 (9.7)
