# Supplementary material for: Bidirectional associations between mental health conditions and cognitive impairment in patients with pain conditions of the back, neck, and spine: A population-based study
Source: PLoS One. 2026 Jun 23;21(6):e0352339. doi: 10.1371/journal.pone.0352339 (PMC13289910; doi:10.1371/journal.pone.0352339)
Supplement: S3 Table — BD: Bipolar Disorder; PTSD: Post-traumatic Stress Disorder; GAD: Generalized Anxiety Disorder; PaD: Panic Disorder; PMD: Persistent Mood disorder; SB: Suicidal Behavior; SCZ: Schizophrenia; SUD: Substance Use Disorder; CKD: Chronic Kidney Disease; CLRD: Chronic Lower Respiratory Disease; CVD: Cardiovascular Diseases; CBVD: Cerebrovascular Diseases; MVC: Metabolic and Vascular Conditions; *: Presented in Number (Percentage of Cohort) format; **: Presented in Mean (Standard Deviation) format. (PDF) [file pone.0352339.s003.pdf]

Table S3. Baseline Demographic Characteristics for Chronic Pain Patients with any mental health condition after Propensity Score Matching. BD: Bipolar Disorder; PTSD: Post-traumatic Stress Disorder; GAD: Generalized Anxiety Disorder; PaD: Panic Disorder; PMD: Persistent Mood disorder; SB: Suicidal Behavior; SCZ: Schizophrenia; SUD: Substance Use Disorder; CKD: Chronic Kidney Disease; CLRD: Chronic Lower Respiratory Disease; CVD: Cardiovascular Diseases; CBVD: Cerebrovascular Diseases; MVC: Metabolic and Vascular Conditions; *: Presented in Number (Percentage of Cohort) format; **: Presented in Mean (Standard Deviation) format.

Characteristic			Control Group	Study Group	Std diff.
Total Population*			466,968 (100)	466,968 (100)	0.333
Age**			67.7 (7.9)	67.3 (7.8)	0.333
Female*			275,508 (59.0)	275,275 (58.9)	0.081
Race*	White		336,005 (72.0)	337,462 (72.3)	0.075
	Black		60,044 (12.9)	59,435 (12.7)	0.072
MVC*	Type 1 Diabetes Mellitus	E10	15,023 (3.2)	14,948 (3.2)	0.142
	Type 2 Diabetes Mellitus	E11	135,397 (29.0)	130,656 (28.0)	0.261
	Overweight and obesity	E66	113,306 (24.3)	107,906 (23.1)	0.327
	Hyperlipidemia	E78	283,473 (60.7)	275,290 (59.0)	0.428
	Essential hypertension	I10	317,207 (39.7)	309,255 (66.2)	0.479
CVD*	Coronary artery/ischemic heart disease	I25	230,326 (12.5)	111,315 (22.6)	0.269
		Z95.1	39,205 (2.1)	18,490 (3.8)	0.097
	Acute myocardial infarction	I21	21,073 (4.5)	21,608 (4.6)	0.166
	Heart failure	I50	50,819 (10.9)	49,848 (10.7)	0.250
	Atrial fibrillation/flutter	I48	49,379 (10.6)	46,824 (10.0)	0.113
	Peripheral arterial disease	I70	67,119 (3.6)	36,805 (7.5)	0.168
		Z95.820	2,064 (0.1)	2,139 (0.4)	0.062
CBVD*	Ischaemic stroke	I63	23,302 (5.0)	22,959 (4.9)	0.161
	Haemorrhagic stroke	I60	2,638 (0.1)	1,725 (0.4)	0.042
		I61	4,350 (0.2)	2,588 (0.5)	0.047
	Transient ischaemic attack	G45	15,311 (3.3)	15,022 (3.2)	0.105
	Other cerebrovascular disease	I67	20,625 (4.4)	20,361 (4.4)	0.143
CLRD*		J40-J47	152,843 (32.7)	152,988 (32.8)	0.550
CKD*		N18	59,021 (12.6)	55,543 (11.9)	0.194
Sepsis*		A40	1,404 (0.1)	1,343 (0.3)	0.047
		A41	28,901 (1.6)	25,212 (5.1)	0.199
