# Supplementary material for: Bidirectional associations between mental health conditions and cognitive impairment in patients with pain conditions of the back, neck, and spine: A population-based study
Source: PLoS One. 2026 Jun 23;21(6):e0352339. doi: 10.1371/journal.pone.0352339 (PMC13289910; doi:10.1371/journal.pone.0352339)
Supplement: S4 Table — BD: Bipolar Disorder; PTSD: Post-traumatic Stress Disorder; GAD: Generalized Anxiety Disorder; PaD: Panic Disorder; PMD: Persistent Mood disorder; SB: Suicidal Behavior; SCZ: Schizophrenia; SUD: Substance Use Disorder; CKD: Chronic Kidney Disease; CLRD: Chronic Lower Respiratory Disease; CVD: Cardiovascular Diseases; CBVD: Cerebrovascular Diseases; MVC: Metabolic and vascular Conditions; *: Presented in Number (Percentage of Cohort) format; **: Presented in Mean (Standard Deviation) format. (PDF) [file pone.0352339.s004.pdf]

Table S4. Baseline Demographic Characteristics for Patients with pain conditions with Depression after Propensity Score Matching. BD: Bipolar Disorder; PTSD: Post-traumatic Stress Disorder; GAD: Generalized Anxiety Disorder; PaD: Panic Disorder; PMD: Persistent Mood disorder; SB: Suicidal Behavior; SCZ: Schizophrenia; SUD: Substance Use Disorder; CKD: Chronic Kidney Disease; CLRD: Chronic Lower Respiratory Disease; CVD: Cardiovascular Diseases; CBVD: Cerebrovascular Diseases; MVC: Metabolic and vascular Conditions; *: Presented in Number (Percentage of Cohort) format; **: Presented in Mean (Standard Deviation) format.

Characteristic			Control Group	Study Group	Std diff.
Total Population*			255,566 (100)	255,566 (100)	0.035
Age**			68.2 (8.1)	68.0 (8.1)	0.035
Female*			175,435 (68.6)	175,011 (68.5)	0.004
Race*	White		193,929 (75.9)	194,061 (75.9)	0.001
	Black		24,893 (9.6)	24,893 (9.7)	0.006
MVC*	Type 1 Diabetes Mellitus	E10	10,499 (4.1)	10,667 (4.2)	0.003
	Type 2 Diabetes Mellitus	E11	81,998 (31.1)	80,362 (31.4)	0.014
	Overweight and obesity	E66	74,953 (29.3)	73,197 (28.6)	0.015
	Hyperlipidemia	E78	170,007 (65.5)	167,458 (65.5)	0.021
	Essential hypertension	I10	181,207 (70.9)	178,626 (69.9)	0.022
CVD*	Coronary artery/ischemic heart disease	I25	56,093 (21.9)	56,131 (22.0)	<0.001
		Z95.1	9,220 (3.6)	9,494 (3.7)	0.006
	Acute myocardial infarction	I21	11,948 (4.7)	12,620 (4.9)	0.012
	Heart failure	I50	32,462 (12.7)	32,380 (12.7)	0.001
	Atrial fibrillation/flutter	I48	29,630 (11.6)	28,754 (11.3)	0.011
	Peripheral arterial disease	I70	16,486 (6.5)	16,983 (6.6)	0.008
		Z95.820	645 (0.3)	852 (0.3)	0.015
CBVD*	Ischaemic stroke	I63	14,341 (5.6)	14,153 (5.5)	0.003
	Haemorrhagic stroke	I60	620 (0.2)	900 (0.4)	0.020
		I61	1,101 (0.4)	1,360 (0.5)	0.015
	Transient ischaemic attack	G45	9,778 (3.8)	9,642 (3.8)	0.003
	Other cerebrovascular disease	I67	12,874 (5.0)	13,060 (5.1)	0.003
CLRD*		J40-J47	87,590 (34.3)	88,068 (34.5)	0.004
CKD*		N18	36,787 (14.4)	35,858 (14.0)	0.010
Sepsis*		A40	529 (0.2)	593 (0.2)	0.005
		A41	12,112 (4.7)	12,087 (4.7)	<0.001
