# Supplementary material for: Bidirectional associations between mental health conditions and cognitive impairment in patients with pain conditions of the back, neck, and spine: A population-based study
Source: PLoS One. 2026 Jun 23;21(6):e0352339. doi: 10.1371/journal.pone.0352339 (PMC13289910; doi:10.1371/journal.pone.0352339)
Supplement: S5 Table — BD: Bipolar Disorder; PTSD: Post-traumatic Stress Disorder; GAD: Generalized Anxiety Disorder; PaD: Panic Disorder; PMD: Persistent Mood disorder; SB: Suicidal Behavior; SCZ: Schizophrenia; SUD: Substance Use Disorder; CKD: Chronic Kidney Disease; CLRD: Chronic Lower Respiratory Disease; CVD: Cardiovascular Diseases; CBVD: Cerebrovascular Diseases; MVC: Metabolic and vascular Conditions; *: Presented in Number (Percentage of Cohort) format; **: Presented in Mean (Standard Deviation) format. (PDF) [file pone.0352339.s005.pdf]

Table S5. Baseline Demographic Characteristics for Patients with pain conditions with Bipolar Disorder after Propensity Score Matching. BD: Bipolar Disorder; PTSD: Post-traumatic Stress Disorder; GAD: Generalized Anxiety Disorder; PaD: Panic Disorder; PMD: Persistent Mood disorder; SB: Suicidal Behavior; SCZ: Schizophrenia; SUD: Substance Use Disorder; CKD: Chronic Kidney Disease; CLRD: Chronic Lower Respiratory Disease; CVD: Cardiovascular Diseases; CBVD: Cerebrovascular Diseases; MVC: Metabolic and vascular Conditions; *: Presented in Number (Percentage of Cohort) format; **: Presented in Mean (Standard Deviation) format.

Characteristic			Control Group	Study Group	Std diff.
Total Population*			23,879 (100)	23,879 (100)	0.033
Age**			64.8 (6.3)	64.6 (6.2)	0.033
Female*			15,346 (64.3)	15,367 (64.4)	0.002
Race*	White		17,427 (73.0)	17,516 (73.4)	0.008
	Black		3,451 (14.5)	3,311 (13.9)	0.017
MVC*	Type 1 Diabetes Mellitus	E10	1,020 (4.3)	1,007 (4.2)	0.003
	Type 2 Diabetes Mellitus	E11	8,107 (34.0)	7,733 (32.4)	0.033
	Overweight and obesity	E66	7,012 (29.4)	6,800 (28.5)	0.020
	Hyperlipidemia	E78	13,890 (58.2)	13,640 (57.1)	0.021
	Essential hypertension	I10	15,769 (66.0)	15,503 (64.9)	0.023
CVD*	Coronary artery/ischemic heart disease	I25	4,830 (20.2)	4,755 (19.9)	0.008
		Z95.1	581 (2.4)	657 (2.8)	0.020
	Acute myocardial infarction	I21	1,036 (4.3)	1,079 (4.5)	0.009
	Heart failure	I50	3,036 (12.7)	2,906 (12.2)	0.016
	Atrial fibrillation/flutter	I48	1,997 (8.4)	1,942 (8.1)	0.008
	Peripheral arterial disease	I70	1,308 (5.5)	1,305 (5.5)	0.001
		Z95.820	57 (0.2)	59 (0.2)	0.002
CBVD*	Ischaemic stroke	I63	1,266 (5.3)	1,266 (5.3)	<0.001
	Haemorrhagic stroke	I60	74 (0.3)	79 (0.3)	0.004
		I61	85 (0.4)	94 (0.4)	0.006
	Transient ischaemic attack	G45	861 (3.6)	868 (3.6)	0.002
	Other cerebrovascular disease	I67	1,258 (5.3)	1,248 (5.2)	0.002
CLRD*		J40-J47	9,652 (40.4)	9,634 (40.3)	0.002
CKD*		N18	3,928 (16.4)	3,548 (14.9)	0.044
Sepsis*		A40	61 (0.3)	61 (0.3)	<0.001
		A41	1,504 (6.3)	1,482 (6.2)	0.004
