# Supplementary material for: Bidirectional associations between mental health conditions and cognitive impairment in patients with pain conditions of the back, neck, and spine: A population-based study
Source: PLoS One. 2026 Jun 23;21(6):e0352339. doi: 10.1371/journal.pone.0352339 (PMC13289910; doi:10.1371/journal.pone.0352339)
Supplement: S6 Table — BD: Bipolar Disorder; PTSD: Post-traumatic Stress Disorder; GAD: Generalized Anxiety Disorder; PaD: Panic Disorder; PMD: Persistent Mood disorder; SB: Suicidal Behavior; SCZ: Schizophrenia; SUD: Substance Use Disorder; CKD: Chronic Kidney Disease; CLRD: Chronic Lower Respiratory Disease; CVD: Cardiovascular Diseases; CBVD: Cerebrovascular Diseases; MVC: Metabolic and vascular Conditions; *: Presented in Number (Percentage of Cohort) format; **: Presented in Mean (Standard Deviation) format. (PDF) [file pone.0352339.s006.pdf]

Table S6. Baseline Demographic Characteristics for Patients with pain conditions with Generalized Anxiety Disorder after Propensity Score Matching. BD: Bipolar Disorder; PTSD: Post-traumatic Stress Disorder; GAD: Generalized Anxiety Disorder; PaD: Panic Disorder; PMD: Persistent Mood disorder; SB: Suicidal Behavior; SCZ: Schizophrenia; SUD: Substance Use Disorder; CKD: Chronic Kidney Disease; CLRD: Chronic Lower Respiratory Disease; CVD: Cardiovascular Diseases; CBVD: Cerebrovascular Diseases; MVC: Metabolic and vascular Conditions; *: Presented in Number (Percentage of Cohort) format; **: Presented in Mean (Standard Deviation) format.

**Characteristic**			**Control Group**	**Study Group**	**Std diff.**
Total Population*			60,166 (100)	60,166 (100)	0.017
Age**			67.9 (8.2)	67.8 (8.2)	0.017
Female*			41,316 (68.7)	41,251 (68.6)	0.002
Race*	White		45,690 (75.9)	45,722 (76.0)	0.001
	Black		4,108 (6.8)	4,247 (7.1)	0.009
MVC*	Type 1 Diabetes Mellitus	E10	2,508 (4.2)	2,529 (4.2)	0.002
	Type 2 Diabetes Mellitus	E11	15,863 (26.4)	15,755 (26.2)	0.004
	Overweight and obesity	E66	15,377 (25.6)	15,249 (25.3)	0.005
	Hyperlipidemia	E78	42,141 (70.0)	41,700 (69.3)	0.016
	Essential hypertension	I10	42,517 (70.7)	42,281 (70.3)	0.009
CVD*	Coronary artery/ischemic heart disease	I25	11,898 (19.8)	12,165 (20.2)	0.011
		Z95.1	1,663 (2.8)	1,871 (3.1)	0.020
	Acute myocardial infarction	I21	2,300 (3.8)	2,540 (4.2)	0.020
	Heart failure	I50	6,219 (10.3)	6,418 (10.7)	0.011
	Atrial fibrillation/flutter	I48	5,963 (9.9)	6,034 (10.0)	0.004
	Peripheral arterial disease	I70	4,033 (6.7)	4,126 (6.9)	0.006
		Z95.820	147 (0.2)	175 (0.3)	0.009
CBVD*	Ischaemic stroke	I63	2,943 (4.9)	3,064 (5.1)	0.009
	Haemorrhagic stroke	I60	123 (0.2)	156 (0.3)	0.011
		I61	193 (0.3)	213 (0.4)	0.006
	Transient ischaemic attack	G45	2,596 (4.3)	2,640 (4.4)	0.004
	Other cerebrovascular disease	I67	3,214 (5.3)	3,269 (5.4)	0.004
CLRD*		J40-J47	21,344 (35.5)	21,422 (35.6)	0.003
CKD*		N18	7,548 (12.5)	7,367 (12.2)	0.009
Sepsis*		A40	86 (0.1)	112 (0.2)	0.011
		A41	2,387 (4.0)	2,440 (4.1)	0.004
