# Supplementary material for: Bidirectional associations between mental health conditions and cognitive impairment in patients with pain conditions of the back, neck, and spine: A population-based study
Source: PLoS One. 2026 Jun 23;21(6):e0352339. doi: 10.1371/journal.pone.0352339 (PMC13289910; doi:10.1371/journal.pone.0352339)
Supplement: S7 Table — BD: Bipolar Disorder; PTSD: Post-traumatic Stress Disorder; GAD: Generalized Anxiety Disorder; PaD: Panic Disorder; PMD: Persistent Mood disorder; SB: Suicidal Behavior; SCZ: Schizophrenia; SUD: Substance Use Disorder; CKD: Chronic Kidney Disease; CLRD: Chronic Lower Respiratory Disease; CVD: Cardiovascular Diseases; CBVD: Cerebrovascular Diseases; MVC: Metabolic and vascular Conditions; *: Presented in Number (Percentage of Cohort) format; **: Presented in Mean (Standard Deviation) format. (PDF) [file pone.0352339.s007.pdf]

Table S7. Baseline Demographic Characteristics for Patients with pain conditions with Post-Traumatic Stress Disorder after Propensity Score Matching. BD: Bipolar Disorder; PTSD: Post-traumatic Stress Disorder; GAD: Generalized Anxiety Disorder; PaD: Panic Disorder; PMD: Persistent Mood disorder; SB: Suicidal Behavior; SCZ: Schizophrenia; SUD: Substance Use Disorder; CKD: Chronic Kidney Disease; CLRD: Chronic Lower Respiratory Disease; CVD: Cardiovascular Diseases; CBVD: Cerebrovascular Diseases; MVC: Metabolic and vascular Conditions; *: Presented in Number (Percentage of Cohort) format; **: Presented in Mean (Standard Deviation) format.

**Characteristic**			**Control Group**	**Study Group**	**Std diff.**
Total Population*			13,545 (100)	13,545 (100)	0.014
Age**			64.9 (6.3)	64.8 (6.1)	0.014
Female*			7,592 (56.1)	7,445 (55.0)	0.022
Race*	White		9,991 (73.8)	10,036 (74.1)	0.008
	Black		1,691 (12.5)	1,667 (12.3)	0.005
MVC*	Type 1 Diabetes Mellitus	E10	500 (3.7)	509 (3.8)	0.004
	Type 2 Diabetes Mellitus	E11	4,371 (32.3)	4,247 (31.4)	0.020
	Overweight and obesity	E66	3,986 (29.4)	3,873 (28.6)	0.018
	Hyperlipidemia	E78	7,990 (59.0)	7,866 (58.1)	0.019
	Essential hypertension	I10	8,945 (66.0)	8,783 (64.8)	0.025
CVD*	Coronary artery/ischemic heart disease	I25	3,120 (23.0)	3,115 (23.0)	0.001
		Z95.1	450 (3.3)	470 (3.5)	0.008
	Acute myocardial infarction	I21	744 (5.5)	762 (5.6)	0.006
	Heart failure	I50	1,646 (12.2)	1,610 (11.9)	0.008
	Atrial fibrillation/flutter	I48	1,328 (9.8)	1,320 (9.7)	0.002
	Peripheral arterial disease	I70	659 (5.0)	678 (4.9)	0.006
		Z95.820	36 (0.3)	51 (0.4)	0.020
CBVD*	Ischaemic stroke	I63	705 (5.2)	745 (5.5)	0.013
	Haemorrhagic stroke	I60	40 (0.3)	54 (0.4)	0.018
		I61	57 (0.4)	54 (0.4)	0.003
	Transient ischaemic attack	G45	443 (3.3)	484 (3.6)	0.017
	Other cerebrovascular disease	I67	591 (4.4)	618 (4.6)	0.010
CLRD*		J40-J47	5,337 (39.4)	5,341 (39.4)	0.001
CKD*		N18	1,727 (12.8)	1,619 (12.0)	0.024
Sepsis*		A40	36 (0.3)	42 (0.3)	0.008
		A41	657 (4.9)	672 (5.0)	0.005
