# Supplementary material for: Bidirectional associations between mental health conditions and cognitive impairment in patients with pain conditions of the back, neck, and spine: A population-based study
Source: PLoS One. 2026 Jun 23;21(6):e0352339. doi: 10.1371/journal.pone.0352339 (PMC13289910; doi:10.1371/journal.pone.0352339)
Supplement: S8 Table — BD: Bipolar Disorder; PTSD: Post-traumatic Stress Disorder; GAD: Generalized Anxiety Disorder; PaD: Panic Disorder; PMD: Persistent Mood disorder; SB: Suicidal Behavior; SCZ: Schizophrenia; SUD: Substance Use Disorder; CKD: Chronic Kidney Disease; CLRD: Chronic Lower Respiratory Disease; CVD: Cardiovascular Diseases; CBVD: Cerebrovascular Diseases; MVC: Metabolic and vascular Conditions; *: Presented in Number (Percentage of Cohort) format; **: Presented in Mean (Standard Deviation) format. (PDF) [file pone.0352339.s008.pdf]

Table S8. Baseline Demographic Characteristics for Patients with pain conditions with Panic Disorder after Propensity Score Matching. BD: Bipolar Disorder; PTSD: Post-traumatic Stress Disorder; GAD: Generalized Anxiety Disorder; PaD: Panic Disorder; PMD: Persistent Mood disorder; SB: Suicidal Behavior; SCZ: Schizophrenia; SUD: Substance Use Disorder; CKD: Chronic Kidney Disease; CLRD: Chronic Lower Respiratory Disease; CVD: Cardiovascular Diseases; CBVD: Cerebrovascular Diseases; MVC: Metabolic and vascular Conditions; *: Presented in Number (Percentage of Cohort) format; **: Presented in Mean (Standard Deviation) format.

Characteristic			Control Group	Study Group	Std diff.
Total Population*			11,146 (100)	11,146 (100)	0.027
Age**			66.5 (7.6)	66.3 (7.5)	0.027
Female*			7,758 (69.6)	7,724 (69.3)	0.007
Race*	White		8,520 (76.4)	8,553 (76.7)	0.007
	Black		929 (8.3)	913 (8.2)	0.005
MVC*	Type 1 Diabetes Mellitus	E10	381 (3.4)	389 (3.5)	0.004
	Type 2 Diabetes Mellitus	E11	3,050 (27.4)	2,989 (26.8)	0.012
	Overweight and obesity	E66	3,273 (29.4)	3,210 (28.8)	0.012
	Hyperlipidemia	E78	7,486 (67.2)	7,373 (66.1)	0.022
	Essential hypertension	I10	7,592 (68.1)	7,504 (67.3)	0.017
CVD*	Coronary artery/ischemic heart disease	I25	2,424 (21.7)	2,426 (21.8)	<0.001
		Z95.1	346 (3.1)	365 (3.3)	0.010
	Acute myocardial infarction	I21	596 (5.3)	655 (5.9)	0.023
	Heart failure	I50	1,415 (12.7)	1,386 (12.4)	0.008
	Atrial fibrillation/flutter	I48	1,190 (10.7)	1,180 (10.6)	0.003
	Peripheral arterial disease	I70	738 (6.6)	727 (6.5)	0.004
		Z95.820	48 (0.4)	52 (0.5)	0.005
CBVD*	Ischaemic stroke	I63	530 (4.8)	557 (5.0)	0.011
	Haemorrhagic stroke	I60	25 (0.2)	33 (0.3)	0.014
		I61	38 (0.3)	47 (0.4)	0.013
	Transient ischaemic attack	G45	531 (4.8)	517 (4.6)	0.006
	Other cerebrovascular disease	I67	624 (5.6)	609 (5.5)	0.006
CLRD*		J40-J47	4,268 (38.3)	4,241 (38.0)	0.005
CKD*		N18	1,371 (12.3)	1,315 (11.8)	0.015
Sepsis*		A40	12 (0.1)	19 (0.2)	0.017
		A41	564 (5.1)	539 (4.8)	0.010
