# Supplementary material for: Bidirectional associations between mental health conditions and cognitive impairment in patients with pain conditions of the back, neck, and spine: A population-based study
Source: PLoS One. 2026 Jun 23;21(6):e0352339. doi: 10.1371/journal.pone.0352339 (PMC13289910; doi:10.1371/journal.pone.0352339)
Supplement: S9 Table — BD: Bipolar Disorder; PTSD: Post-traumatic Stress Disorder; GAD: Generalized Anxiety Disorder; PaD: Panic Disorder; PMD: Persistent Mood disorder; SB: Suicidal Behavior; SCZ: Schizophrenia; SUD: Substance Use Disorder; CKD: Chronic Kidney Disease; CLRD: Chronic Lower Respiratory Disease; CVD: Cardiovascular Diseases; CBVD: Cerebrovascular Diseases; MVC: Metabolic and vascular Conditions; *: Presented in Number (Percentage of Cohort) format; **: Presented in Mean (Standard Deviation) format. (PDF) [file pone.0352339.s009.pdf]

**Table S9. Baseline Demographic Characteristics for Patients with pain conditions with Persistent Mood Disorder after Propensity Score Matching.** BD: Bipolar Disorder; PTSD: Post-traumatic Stress Disorder; GAD: Generalized Anxiety Disorder; PaD: Panic Disorder; PMD: Persistent Mood disorder; SB: Suicidal Behavior; SCZ: Schizophrenia; SUD: Substance Use Disorder; CKD: Chronic Kidney Disease; CLRD: Chronic Lower Respiratory Disease; CVD: Cardiovascular Diseases; CBVD: Cerebrovascular Diseases; MVC: Metabolic and vascular Conditions; *: Presented in Number (Percentage of Cohort) format; **: Presented in Mean (Standard Deviation) format.

| Characteristic | | | Control Group | Study Group | Std diff. |
|---|---|---|---|---|---|
| Total Population* | | | 14,746 (100) | 14,746 (100) | 0.014 |
| Age** | | | 67.3 (7.9) | 67.2 (8.0) | 0.014 |
| Female* | | | 9,550 (64.8) | 9,522 (64.6) | 0.004 |
| Race* | White | | 10,230 (69.4) | 10,255 (69.5) | 0.004 |
| | Black | | 1,093 (7.4) | 1,144 (7.8) | 0.013 |
| MVC* | Type 1 Diabetes Mellitus | E10 | 879 (6.0) | 879 (6.0) | <0.001 |
| | Type 2 Diabetes Mellitus | E11 | 4,269 (29.0) | 4,216 (28.6) | 0.008 |
| | Overweight and obesity | E66 | 4,637 (31.4) | 4,584 (31.1) | 0.008 |
| | Hyperlipidemia | E78 | 10,543 (71.5) | 10,429 (70.7) | 0.017 |
| | Essential hypertension | I10 | 10,243 (69.5) | 10,215 (69.3) | 0.004 |
| CVD* | Coronary artery/ischemic heart disease | I25 | 2,820 (19.1) | 2,904 (19.7) | 0.014 |
| | | Z95.1 | 367 (2.5) | 445 (3.0) | 0.032 |
| | Acute myocardial infarction | I21 | 617 (4.2) | 662 (4.5) | 0.015 |
| | Heart failure | I50 | 1,418 (9.6) | 1,516 (10.3) | 0.022 |
| | Atrial fibrillation/flutter | I48 | 1,349 (9.1) | 1,393 (9.4) | 0.010 |
| | Peripheral arterial disease | I70 | 908 (6.2) | 941 (6.4) | 0.009 |
| | | Z95.820 | 19 (0.1) | 24 (0.2) | 0.009 |
| CBVD* | Ischaemic stroke | I63 | 811 (5.5) | 839 (5.7) | 0.008 |
| | Haemorrhagic stroke | I60 | 46 (0.3) | 57 (0.4) | 0.013 |
| | | I61 | 60 (0.4) | 56 (0.4) | 0.004 |
| | Transient ischaemic attack | G45 | 681 (4.6) | 699 (4.7) | 0.006 |
| | Other cerebrovascular disease | I67 | 990 (6.7) | 1,030 (7.0) | 0.011 |
| CLRD* | | J40-J47 | 5,163 (35.0) | 5,190 (35.2) | 0.004 |
| CKD* | | N18 | 1,824 (12.4) | 1,794 (12.2) | 0.006 |
| Sepsis* | | A40 | 30 (0.2) | 33 (0.2) | 0.004 |
| | | A41 | 504 (3.4) | 569 (3.9) | 0.024 |
