# Supplementary material for: Bidirectional associations between mental health conditions and cognitive impairment in patients with pain conditions of the back, neck, and spine: A population-based study
Source: PLoS One. 2026 Jun 23;21(6):e0352339. doi: 10.1371/journal.pone.0352339 (PMC13289910; doi:10.1371/journal.pone.0352339)
Supplement: S10 Table — BD: Bipolar Disorder; PTSD: Post-traumatic Stress Disorder; GAD: Generalized Anxiety Disorder; PaD: Panic Disorder; PMD: Persistent Mood disorder; SB: Suicidal Behavior; SCZ: Schizophrenia; SUD: Substance Use Disorder; CKD: Chronic Kidney Disease; CLRD: Chronic Lower Respiratory Disease; CVD: Cardiovascular Diseases; CBVD: Cerebrovascular Diseases; MVC: Metabolic and vascular Conditions; *: Presented in Number (Percentage of Cohort) format; **: Presented in Mean (Standard Deviation) format. (PDF) [file pone.0352339.s010.pdf]

Table S10. Baseline Demographic Characteristics for Patients with pain conditions with Suicidal Behavior after Propensity Score Matching. BD: Bipolar Disorder; PTSD: Post-traumatic Stress Disorder; GAD: Generalized Anxiety Disorder; PaD: Panic Disorder; PMD: Persistent Mood disorder; SB: Suicidal Behavior; SCZ: Schizophrenia; SUD: Substance Use Disorder; CKD: Chronic Kidney Disease; CLRD: Chronic Lower Respiratory Disease; CVD: Cardiovascular Diseases; CBVD: Cerebrovascular Diseases; MVC: Metabolic and vascular Conditions; *: Presented in Number (Percentage of Cohort) format; **: Presented in Mean (Standard Deviation) format.

Characteristic			Control Group	Study Group	Std diff.
Total Population*			7,329 (100)	7,329 (100)	0.023
Age**			65.0 (7.0)	64.8 (7.0)	0.023
Female*			3,524 (48.1)	3,504 (47.8)	0.005
Race*	White		5,322 (72.6)	5,325 (72.7)	0.001
	Black		1,148 (15.7)	1,104 (15.1)	0.017
MVC*	Type 1 Diabetes Mellitus	E10	289 (3.9)	284 (3.9)	0.004
	Type 2 Diabetes Mellitus	E11	2,293 (31.3)	2,228 (30.4)	0.019
	Overweight and obesity	E66	1,579 (21.5)	1,543 (21.1)	0.012
	Hyperlipidemia	E78	3,514 (47.9)	3,497 (47.7)	0.005
	Essential hypertension	I10	4,909 (67.0)	4,833 (65.9)	0.022
CVD*	Coronary artery/ischemic heart disease	I25	1,723 (23.5)	1,695 (23.1)	0.009
		Z95.1	291 (4.0)	293 (4.0)	0.001
	Acute myocardial infarction	I21	423 (5.8)	462 (6.3)	0.022
	Heart failure	I50	1,024 (14.0)	1,016 (13.9)	0.003
	Atrial fibrillation/flutter	I48	797 (10.9)	749 (10.2)	0.021
	Peripheral arterial disease	I70	399 (5.4)	418 (5.7)	0.011
		Z95.820	24 (0.3)	28 (0.4)	0.009
CBVD*	Ischaemic stroke	I63	427 (5.8)	437 (6.0)	0.006
	Haemorrhagic stroke	I60	19 (0.3)	26 (0.4)	0.017
		I61	42 (0.6)	40 (0.5)	0.004
	Transient ischaemic attack	G45	198 (2.7)	206 (2.8)	0.007
	Other cerebrovascular disease	I67	416 (5.7)	416 (5.7)	<0.001
CLRD*		J40-J47	2,662 (36.3)	2,633 (35.9)	0.008
CKD*		N18	960 (13.1)	953 (13.0)	0.003
Sepsis*		A40	29 (0.4)	33 (0.5)	0.008
		A41	560 (7.6)	540 (7.4)	0.010
