# Supplementary material for: Bidirectional associations between mental health conditions and cognitive impairment in patients with pain conditions of the back, neck, and spine: A population-based study
Source: PLoS One. 2026 Jun 23;21(6):e0352339. doi: 10.1371/journal.pone.0352339 (PMC13289910; doi:10.1371/journal.pone.0352339)
Supplement: S11 Table — BD: Bipolar Disorder; PTSD: Post-traumatic Stress Disorder; GAD: Generalized Anxiety Disorder; PaD: Panic Disorder; PMD: Persistent Mood disorder; SB: Suicidal Behavior; SCZ: Schizophrenia; SUD: Substance Use Disorder; CKD: Chronic Kidney Disease; CLRD: Chronic Lower Respiratory Disease; CVD: Cardiovascular Diseases; CBVD: Cerebrovascular Diseases; MVC: Metabolic and vascular Conditions; *: Presented in Number (Percentage of Cohort) format; **: Presented in Mean (Standard Deviation) format. (PDF) [file pone.0352339.s011.pdf]

Table S11. Baseline Demographic Characteristics for Patients with pain conditions with Schizophrenia after Propensity Score Matching. BD: Bipolar Disorder; PTSD: Post-traumatic Stress Disorder; GAD: Generalized Anxiety Disorder; PaD: Panic Disorder; PMD: Persistent Mood disorder; SB: Suicidal Behavior; SCZ: Schizophrenia; SUD: Substance Use Disorder; CKD: Chronic Kidney Disease; CLRD: Chronic Lower Respiratory Disease; CVD: Cardiovascular Diseases; CBVD: Cerebrovascular Diseases; MVC: Metabolic and vascular Conditions; *: Presented in Number (Percentage of Cohort) format; **: Presented in Mean (Standard Deviation) format.

Characteristic			Control Group	Study Group	Std diff.
Total Population*			15,533 (100)	15,533 (100)	0.039
Age**			66.2 (7.5)	66.0 (7.5)	0.039
Female*			8,245 (53.1)	8,303 (53.5)	0.007
Race*	White		8,868 (57.1)	8,888 (57.2)	0.003
	Black		4,261 (27.4)	4,130 (26.6)	0.019
MVC*	Type 1 Diabetes Mellitus	E10	852 (5.5)	837 (5.4)	0.004
	Type 2 Diabetes Mellitus	E11	5,938 (38.2)	5,730 (36.9)	0.028
	Overweight and obesity	E66	4,143 (26.7)	4,104 (26.4)	0.006
	Hyperlipidemia	E78	8,709 (56.1)	8,533 (54.9)	0.023
	Essential hypertension	I10	11,065 (71.2)	10,836 (69.8)	0.032
CVD*	Coronary artery/ischemic heart disease	I25	3,390 (21.8)	3,320 (21.4)	0.011
		Z95.1	482 (3.1)	460 (3.0)	0.008
	Acute myocardial infarction	I21	882 (5.7)	905 (5.8)	0.006
	Heart failure	I50	2,596 (16.7)	2,498 (16.1)	0.017
	Atrial fibrillation/flutter	I48	1,688 (10.9)	1,625 (10.5)	0.013
	Peripheral arterial disease	I70	1,073 (6.9)	1,046 (6.7)	0.007
		Z95.820	49 (0.3)	39 (0.3)	0.012
CBVD*	Ischaemic stroke	I63	1,212 (7.8)	1,187 (7.6)	0.006
	Haemorrhagic stroke	I60	53 (0.3)	56 (0.4)	0.003
		I61	87 (0.6)	80 (0.5)	0.006
	Transient ischaemic attack	G45	682 (4.4)	660 (4.2)	0.007
	Other cerebrovascular disease	I67	1,259 (8.1)	1,249 (8.0)	0.002
CLRD*		J40-J47	6,174 (39.7)	6,167 (39.7)	0.001
CKD*		N18	2,792 (18.0)	2,612 (16.8)	0.031
Sepsis*		A40	67 (0.4)	66 (0.4)	0.001
		A41	1,442 (9.3)	1,494 (9.6)	0.011
