# Supplementary material for: Bidirectional associations between mental health conditions and cognitive impairment in patients with pain conditions of the back, neck, and spine: A population-based study
Source: PLoS One. 2026 Jun 23;21(6):e0352339. doi: 10.1371/journal.pone.0352339 (PMC13289910; doi:10.1371/journal.pone.0352339)
Supplement: S12 Table — BD: Bipolar Disorder; PTSD: Post-traumatic Stress Disorder; GAD: Generalized Anxiety Disorder; PaD: Panic Disorder; PMD: Persistent Mood disorder; SB: Suicidal Behavior; SCZ: Schizophrenia; SUD: Substance Use Disorder; CKD: Chronic Kidney Disease; CLRD: Chronic Lower Respiratory Disease; CVD: Cardiovascular Diseases; CBVD: Cerebrovascular Diseases; MVC: Metabolic and vascular Conditions; *: Presented in Number (Percentage of Cohort) format; **: Presented in Mean (Standard Deviation) format. (PDF) [file pone.0352339.s012.pdf]

Table S12. Baseline Demographic Characteristics for Patients with pain conditions with Substance Use Disorder after Propensity Score Matching. BD: Bipolar Disorder; PTSD: Post-traumatic Stress Disorder; GAD: Generalized Anxiety Disorder; PaD: Panic Disorder; PMD: Persistent Mood disorder; SB: Suicidal Behavior; SCZ: Schizophrenia; SUD: Substance Use Disorder; CKD: Chronic Kidney Disease; CLRD: Chronic Lower Respiratory Disease; CVD: Cardiovascular Diseases; CBVD: Cerebrovascular Diseases; MVC: Metabolic and vascular Conditions; *: Presented in Number (Percentage of Cohort) format; **: Presented in Mean (Standard Deviation) format.

Characteristic			Control Group	Study Group	Std diff.
Total Population*			214,545 (100)	214,545 (100)	0.038
Age**			65.9 (7.0)	65.6 (6.8)	0.038
Female*			99,927 (46.6)	100,779 (47.0)	0.008
Race*	White		145,426 (67.8)	146,302 (68.2)	0.009
	Black		38,579 (18.0)	37,801 (17.6)	0.009
MVC*	Type 1 Diabetes Mellitus	E10	6,694 (3.1)	6,251 (2.9)	0.012
	Type 2 Diabetes Mellitus	E11	60,302 (28.1)	57,249 (26.7)	0.032
	Overweight and obesity	E66	44,737 (20.9)	42,177 (19.7)	0.030
	Hyperlipidemia	E78	115,806 (54.0)	113,167 (52.7)	0.025
	Essential hypertension	I10	143,796 (67.0)	139,979 (65.2)	0.038
CVD*	Coronary artery/ischemic heart disease	I25	51,712 (24.1)	50,263 (23.4)	0.016
		Z95.1	8,435 (3.9)	8,262 (3.9)	0.004
	Acute myocardial infarction	I21	12,467 (5.8)	12,522 (5.8)	0.001
	Heart failure	I50	26,077 (12.2)	25,139 (11.7)	0.013
	Atrial fibrillation/flutter	I48	22,242 (10.4)	20,688 (9.6)	0.024
	Peripheral arterial disease	I70	18,059 (8.4)	17,523 (8.2)	0.009
		Z95.820	792 (0.4)	1,123 (0.5)	0.023
CBVD*	Ischaemic stroke	I63	12,139 (5.7)	11,677 (5.4)	0.009
	Haemorrhagic stroke	I60	627 (0.3)	780 (0.4)	0.012
		I61	985 (0.5)	1,096 (0.5)	0.007
	Transient ischaemic attack	G45	6,583 (3.1)	6,473 (3.0)	0.003
	Other cerebrovascular disease	I67	9,796 (4.6)	9,452 (4.4)	0.008
CLRD*		J40-J47	86,042 (40.1)	85,127 (39.7)	0.009
CKD*		N18	25,402 (11.8)	23,630 (11.0)	0.026
Sepsis*		A40	508 (0.2)	611 (0.3)	0.009
		A41	10,861 (5.1)	10,588 (4.9)	0.006
