# Supplementary material for: Bidirectional associations between mental health conditions and cognitive impairment in patients with pain conditions of the back, neck, and spine: A population-based study
Source: PLoS One. 2026 Jun 23;21(6):e0352339. doi: 10.1371/journal.pone.0352339 (PMC13289910; doi:10.1371/journal.pone.0352339)
Supplement: S13 Table — BD: Bipolar Disorder; PTSD: Post-traumatic Stress Disorder; GAD: Generalized Anxiety Disorder; PaD: Panic Disorder; PMD: Persistent Mood disorder; SB: Suicidal Behavior; SCZ: Schizophrenia; SUD: Substance Use Disorder; CKD: Chronic Kidney Disease; CLRD: Chronic Lower Respiratory Disease; CVD: Cardiovascular Diseases; CBVD: Cerebrovascular Diseases; MVC: Metabolic and vascular Conditions; *: Presented in Number (Percentage of Cohort) format; **: Presented in Mean (Standard Deviation) format. (PDF) [file pone.0352339.s013.pdf]

Table S13. Baseline Demographic Characteristics for Patients with pain conditions with Cognitive Impairment after Propensity Score Matching. BD: Bipolar Disorder; PTSD: Post-traumatic Stress Disorder; GAD: Generalized Anxiety Disorder; PaD: Panic Disorder; PMD: Persistent Mood disorder; SB: Suicidal Behavior; SCZ: Schizophrenia; SUD: Substance Use Disorder; CKD: Chronic Kidney Disease; CLRD: Chronic Lower Respiratory Disease; CVD: Cardiovascular Diseases; CBVD: Cerebrovascular Diseases; MVC: Metabolic and vascular Conditions; *: Presented in Number (Percentage of Cohort) format; **: Presented in Mean (Standard Deviation) format.

**Characteristic**			**Control Group**	**Study Group**	**Std diff.**
Total Population*			36,552 (100)	36,552 (100)	0.013
Age**			82.7 (7.0)	82.6 (7.1)	0.013
Female*			23,130 (63.3)	23,215 (63.5)	0.005
Race*	White		25,394 (69.5)	24,940 (68.2)	0.027
	Black		3,766 (10.3)	4,045 (11.1)	0.025
MVC*	Type 1 Diabetes Mellitus	E10	823 (2.3)	832 (2.3)	0.002
	Type 2 Diabetes Mellitus	E11	10,709 (29.3)	10,717 (29.3)	<0.001
	Overweight and obesity	E66	3,118 (8.5)	3,136 (8.6)	0.002
	Hyperlipidemia	E78	20,293 (55.5)	19,943 (54.6)	0.019
	Essential hypertension	I10	26,329 (72.0)	25,546 (69.9)	0.047
CVD*	Coronary artery/ischemic heart disease	I25	10,520 (28.8)	10,232 (28.0)	0.017
		Z95.1	1,932 (5.3)	1,887 (5.2)	0.006
	Acute myocardial infarction	I21	2,370 (6.5)	2,481 (6.8)	0.012
	Heart failure	I50	7,274 (19.9)	7,339 (20.1)	0.004
	Atrial fibrillation/flutter	I48	9,010 (24.6)	8,770 (24.0)	0.015
	Peripheral arterial disease	I70	3,416 (9.3)	3,414 (9.3)	<0.001
		Z95.820	98 (0.3)	101 (0.3)	0.002
CBVD*	Ischaemic stroke	I63	3,777 (10.3)	4,058 (11.1)	0.025
	Haemorrhagic stroke	I60	140 (0.4)	175 (0.5)	0.015
		I61	364 (1.0)	448 (1.2)	0.022
	Transient ischaemic attack	G45	1,994 (5.5)	2,025 (5.5)	0.004
	Other cerebrovascular disease	I67	4,301 (11.8)	4,581 (12.5)	0.023
CLRD*		J40-J47	7,153 (19.6)	7,025 (19.2)	0.009
CKD*		N18	8,353 (22.9)	8,425 (23.0)	0.005
Sepsis*		A40	118 (0.3)	120 (0.3)	0.001
		A41	3,149 (8.6)	3,437 (9.4)	0.028
